# Requirements for the differentiation of innate T-bet^high^ memory-phenotype CD4^+^ T lymphocytes under steady state

**DOI:** 10.1038/s41467-020-17136-1

**Published:** 2020-07-06

**Authors:** Takeshi Kawabe, Jaeu Yi, Akihisa Kawajiri, Kerry Hilligan, Difeng Fang, Naoto Ishii, Hidehiro Yamane, Jinfang Zhu, Dragana Jankovic, Kwang Soon Kim, Giorgio Trinchieri, Alan Sher

**Affiliations:** 1 Immunobiology Section, Laboratory of Parasitic Diseases, National Institute of Allergy and Infectious Diseases (NIAID), National Institutes of Health (NIH), Bethesda, MD 20892 USA; 2 Department of Microbiology and Immunology, Tohoku University Graduate School of Medicine, Sendai, 980-8575 Japan; 3 Academy of Immunology and Microbiology, Institute for Basic Science, Pohang, 37666 Republic of Korea; 4 Department of Integrative Biosciences and Biotechnology, Pohang University of Science and Technology, Pohang, 37673 Republic of Korea; 5 Molecular and Cellular Immunoregulation Section, Laboratory of Immune System Biology, NIAID, NIH, Bethesda, MD 20892 USA; 6 Cellular Immunology Section, Vaccine Research Center, NIAID, NIH, Bethesda, MD 20892 USA; 7 Cancer and Inflammation Program, Center for Cancer Research, National Cancer Institute, NIH, Bethesda, MD 20892 USA

**Keywords:** Immunological memory, Infection, Innate immune cells, CD4-positive T cells

## Abstract

CD4^+^ T lymphocytes consist of naïve, antigen-specific memory, and memory-phenotype (MP) cell compartments at homeostasis. We recently showed that MP cells exert innate-like effector function during host defense, but whether MP CD4^+^ T cells are functionally heterogeneous and, if so, what signals specify the differentiation of MP cell subpopulations under homeostatic conditions is still unclear. Here we characterize MP lymphocytes as consisting of T-bet^high^, T-bet^low^, and T-bet^−^ subsets, with innate, Th1-like effector activity exclusively associated with T-bet^high^ cells. We further show that the latter population depends on IL-12 produced by CD8α^+^ type 1 dendritic cells (DC1) for its differentiation. Finally, our data demonstrate that this tonic IL-12 production requires TLR-MyD88 signaling independent of foreign agonists, and is further enhanced by CD40-CD40L interactions between DC1 and CD4^+^ T lymphocytes. We propose that optimal differentiation of T-bet^high^ MP lymphocytes at homeostasis is driven by self-recognition signals at both the DC and Tcell levels.

## Abstract (web summary)

CD4^+^ T cells contain a T-bet^high^ memory-phenotype (MP) population with innate-like functions. Here the authors characterize the requirements for their differentiation at homeostasis and identify a function for IL-12 that is tonically produced by type 1 dendritic cells in an MyD88- and CD40-dependent, but foreign PAMP-independent manner.

## Introduction

Conventional CD4^+^ T lymphocytes consist of naïve (CD44^low^CD62L^high^), foreign antigen (Ag)-specific authentic memory, and memory-phenotype (MP; both CD44^high^CD62L^low^) compartments in steady state^1^. While naïve and Ag-specific memory cells are essential in adaptive immune responses against primary and secondary infections, respectively, MP cells can exert rapid and innate-like effector function in Th1-type immunity and later contribute to the development of Ag-specific effector T cells^2^. In this regard, we have previously shown that MP CD4^+^ T cells rapidly produce IFN-γ in response to Th1-associated inflammatory cytokines in the absence of foreign Ag recognition and can participate in host defense against *Toxoplasma* infection^2^. We proposed that this type of innate-like activity exerted by MP cells may significantly contribute to the innate immune resistance mediated by natural killer (NK) cells, innate lymphoid cells (ILCs), and virtual memory CD8^+^ T lymphocytes^3–5^.

Despite the phenotypic similarities between MP and foreign Ag-specific memory CD4^+^ T lymphocytes in terms of CD44 and CD62L expression, the two populations can be distinguished from each other based on other properties. Thus, because MP cells are present at similar levels in specific pathogen-free (SPF), germ-free (GF), and antigen-free (AF) animals that lack virtually all foreign Ags^6,7^, recognition of self Ags is thought to provide the major stimulus for their development in contrast to foreign Ags, which drive conventional memory T cells. In addition, MP cells rapidly proliferate in steady state while conventional memory T lymphocytes are essentially quiescent^8^, suggesting distinct mechanisms for their maintenance as well as function.

MP lymphocytes arise under homeostatic conditions from naïve precursors in a manner dependent on both T cell receptor (TCR) and CD28 signaling^2,9^. These stimuli which serve as signals 1 and 2 for MP generation are proposed to be constantly provided by dendritic cells (DCs) expressing self Ags^10^, and this hypothetical pathway has been partially confirmed in vivo^11,12^.

While the signals driving MP generation have been well studied, it has not been clear whether these cells exist in functionally heterogenous subpopulations as do conventional effector CD4^+^ T lymphocytes and if so, which factors determine their selective differentiation under homeostatic conditions. We previously found that MP cells tonically express T-bet^2^, which is not unexpected since MP cells produce IFN-γ in response to inflammatory cytokines in a manner similar to T-bet- and/or Eomes-expressing NK cells and type 1 ILCs^3,13–16^. Our previous work further indicated that the expression of T-bet in MP cells is dependent on IL-12B p40^2^, but the source of this cytokine and the factors that regulate its production under steady-state conditions were not characterized. In the case of conventional helper T cell differentiation, Ag-specific effector cells differentiate into a T-bet^+^ Th1 subset under the influence of IL-12^17–20^. In this situation, the IL-12 is derived from distinct subsets of DCs in response to microbial-derived components and further upregulated by CD40 signaling^21,22^. Given the aforementioned similarities between foreign Ag-specific memory and MP CD4^+^ T cells, we asked whether an analogous DC-derived signal 3 also plays a role in driving and maintaining T-bet^+^ MP differentiation under steady-state conditions.

In the present study we have characterized the heterogeneity of MP CD4^+^ T cells in steady state in terms of their expression of master transcription factors and, in the case of the T-bet^+^ subpopulation, analyzed the IL-12-mediated mechanisms that promote its differentiation. Our observations reveal a specific role for IL-12 homeostatically produced by CD8α^+^ type 1 DCs (DC1) in the steady-state differentiation of T-bet^high^ MP cells.

## Results

### MP CD4^+^ T cells contain an innate T-bet^high^ subpopulation

**Fig. 1:**
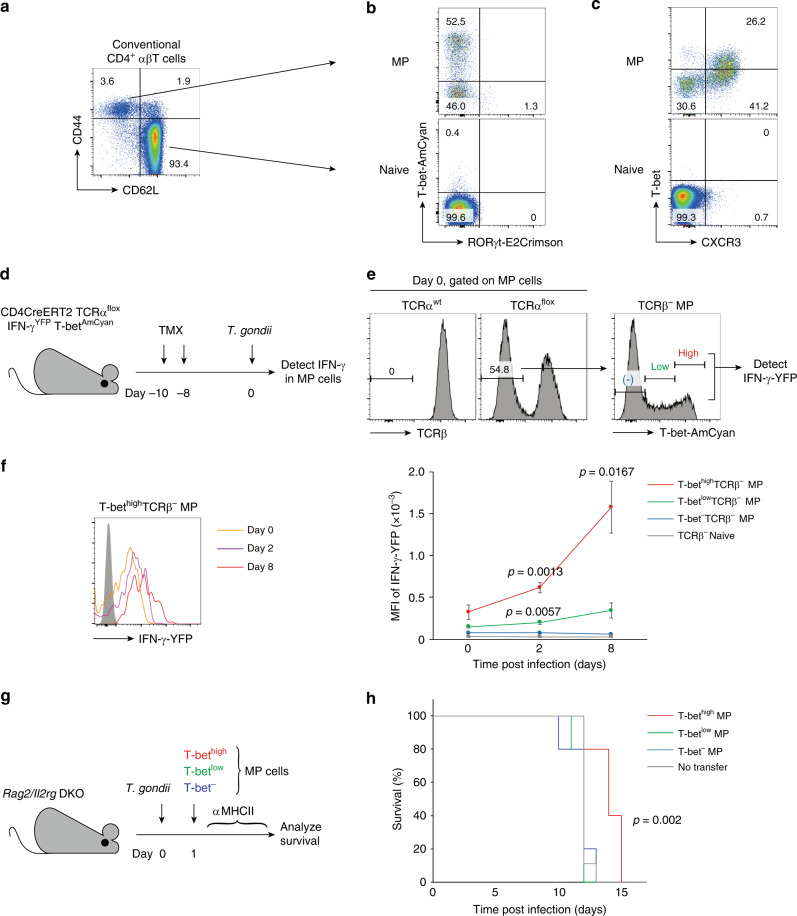
MP CD4^+^ T lymphocytes contain three subsets based on T-bet expression. (**a**) Definition of MP cells. The representative dot plot shows CD44 and CD62L expression by Foxp3^−^CD4^+^ αβT lymphocytes in spleen from five mice. (**b** and **c**) T-bet and RORγt expression in MP and naïve CD4^+^ T lymphocytes. The representative plots show (**b**) T-bet-AmCyan and RORγt-E2Crimson expression in the indicated cell populations obtained from three T-bet-AmCyan RORγt-E2Crimson double reporter mice and (**c**) T-bet and CXCR3 protein levels in the same populations from five mice. (**d**–**f**) T-bet^high^ MP cells produce IFN-γ in the absence of TCR signaling in *Toxoplasma-*infected mice. **d** Experimental design. CD4CreERT2 TCRα^flox^ IFN-γ-YFP T-bet-AmCyan mice receiving TMX on Days -10 and -8 were infected with *T. gondii* on Day 0 and splenocytes measured for IFN-γ-YFP expression in T-bet^high^, T-bet^low^, and T-bet^−^TCRβ^−^ MP subsets several days later. **e** TCRβ expression in MP cells and T-bet-AmCyan levels in their TCRβ^−^ fraction on Day 0. **f** IFN-γ-YFP expression in T-bet^high^, T-bet^low^, and T-bet^−^TCRβ^−^ MP cells on Days 0, 2, and 8 following *T. gondii* infection. A representative histogram is shown (orange, purple, and red lines indicate YFP expression by T-bet^high^TCRβ^−^ MP cells on Days 0, 2, and 8, respectively) while the graph depicts the MFI (mean ± SD) of IFN-γ-YFP expression in the indicated populations (red T-bet^high^TCRβ^−^ MP; green T-bet^low^TCRβ^−^ MP; blue T-bet^−^TCRβ^−^ MP; gray TCRβ^−^ Naïve; Day 0 *n* = 6 mice; Day 2 *n* = 3 mice; Day 8 *n* = 3 mice). Data are representative of two independent experiments performed. (**g** and **h**) T-bet^high^ MP cells can prolong survival in *T. gondii* infection. **g** Experimental design. *Rag2 / Il2rg* DKO mice were infected with *T. gondii* on Day 0, received sorted T-bet^high^, T-bet^low^, or T-bet^−^ MP cells on the next day, and were monitored for survival. **h** Survival of *T. gondii*-infected *Rag2 / Il2rg* DKO mice that received each MP subset (red, green, and blue lines show T-bet^high^, T-bet^low^, and T-bet^−^ MP cells, respectively; T-bet^high^ MP *n* = 5 mice; T-bet^low^ MP *n* = 5 mice; T-bet^−^ MP *n* = 5 mice; Untransferred controls *n* = 9 mice). Data are pooled from two experiments performed. (**f**) A two-sided *t* and (**h**) a log-rank tests were performed to assess significance. Source data are provided as a Source Data file.

As revealed in our previous work^2^, MP CD4^+^ T cells exist under uninfected, steady-state conditions as CD44^high^CD62L^low^Foxp3^−^CD4^+^ αβT lymphocytes in the spleen, a major site of their generation (Fig. 1a; gating strategy is shown in Methods). RNAseq analysis performed in the same study showed that genes associated with Th1 and Th17 but not Th2 differentiation are highly enriched in MP as compared with the naïve CD4^+^ T cells. Consistent with this finding, using unstimulated T-bet-AmCyan RORγt-E2Crimson double reporter mice, we observed that resting state MP lymphocytes are composed of major T-bet^high^ and minor RORγt^+^ subpopulations (Fig. 1b). We quantitated by immunofluorescence the protein expression of T-bet and CXCR3 (a chemokine receptor whose expression is closely associated with the Th1 subset^23^) in MP and naïve CD4^+^ T cells. This analysis confirmed that MP cells consist of T-bet^high^CXCR3^+^, T-bet^low^CXCR3^+^, and T-bet^−^CXCR3^−^ subpopulations while naïve T cells are essentially all T-bet^−^CXCR3^−^ (Fig. 1c). We next used T-bet-ZsGreen reporter mice that express fixable ZsGreen upon activation of *Tbx21* gene promoter to assess *Tbx21* transcription in these three subsets. The MP cells in these animals consisted of three subsets with ZsGreen^high^, ZsGreen^low^, and ZsGreen^−^ largely corresponding to the T-bet^high^CXCR3^+^, T-bet^low^CXCR3^+^, and T-bet^−^CXCR3^−^ MP subpopulations, respectively (Supplementary Fig. 1). On the basis of this evidence as well as the findings in our previous report^2^, we concluded that under homeostatic conditions MP cells consist of T-bet^high^, T-bet^low^, and T-bet^−^ subpopulations in terms of transcription, mRNA accumulation, and protein expression.

In response to infection with Th1-inducing pathogens, MP cells produce IFN-γ in an IL-12-dependent manner in the absence of pathogen Ag recognition^2^. To examine if the T-bet^high^ but no other MP subsets can produce IFN-γ in the absence of conventional TCR signaling, we utilized CD4CreERT2 TCRα^flox^ mice to delete TCR in existing MP cells after their generation and crossed them with T-bet-AmCyan IFN-γ-YFP double reporter mice to efficiently detect IFN-γ in vivo. When these animals were injected twice with tamoxifen (TMX) at a 48 h interval and TCRβ expression examined 10 days after the first injection (referred to as Day 0) (Fig. 1d), ~50% of the MP cells lost TCRβ (Fig. 1e) as previously described in conventional and regulatory T cells^24,25^. This TCRβ^−^ MP cell population still consisted of T-bet^high^, T-bet^low^, and T-bet^−^ subsets (Fig. 1e). We then infected the mice with *Toxoplasma gondii*, which we have previously shown generates an innate IL-12-dependent IFN-γ response by MP cells^2^, on Day 0 and measured IFN-γ produced by T-bet^high^, T-bet^low^, and T-bet^−^TCRβ^−^ MP cells 2 and 8 days later. T-bet^high^ MP cells were found to be the dominant population that produces IFN-γ with T-bet^low^ cells making only a minimal contribution to this response (Fig. 1f). Thus, as expected the T-bet^high^ subset is the major subpopulation of MP cells that produces innate IFN-γ in the absence of conventional TCR signaling.

In *Toxoplasma* infection, MP cells can prolong survival for 2–3 days in an innate-like manner during the critical period before development of adaptive T cell responses^2^. To determine which MP subpopulation contributes to this enhancement of survival, we infected *Rag2/Il2rg* double knockout (DKO) mice with *T. gondii* and subsequently transferred either T-bet^high^, T-bet^low^, or T-bet^−^ MP cells from T-bet reporter mice to the same animals one day later (Fig. 1g). During the course of the experiment, anti-MHC class II (MHCII) monoclonal antibody (mAb) was administered every 3 days to block any foreign Ag-specific MP cell responses as previously described^2^. As predicted, T-bet^high^ but not T-bet^low^ or T-bet^−^ MP cells were found to prolong survival of the infected mice (Fig. 1h). Thus, among the three MP subpopulations, T-bet^high^ cells appear to be the most potent in exerting innate host-protective function.

### Tonic IL-12 from DC1 promotes T-bet^high^ MP differentiation

**Fig. 2:**
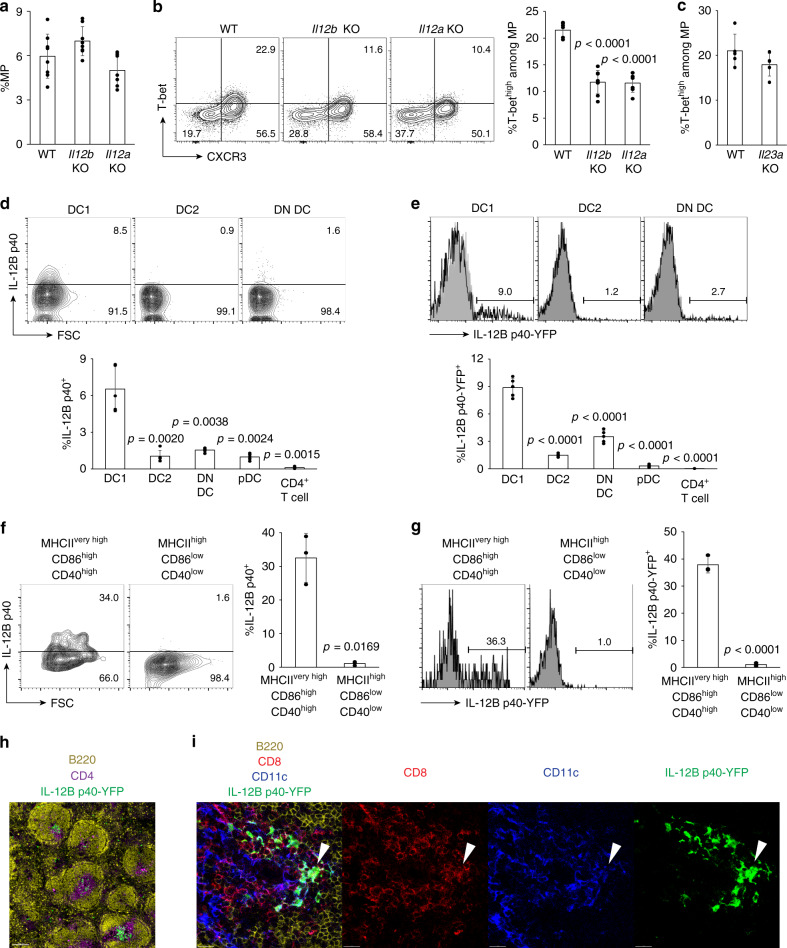
MP lymphocytes differentiate into the T-bet^high^ subset in the presence of DC1-derived IL-12. (**a** and **b**) IL-12 p40 and p35 are essential for optimal MP differentiation. **a** The frequency (mean ± SD) of MP cells among CD4^+^ T lymphocytes obtained from WT, *Il12b* KO, and *Il12a* KO mice is shown (WT *n* = 8 mice; *Il12b* KO *n* = 8 mice; *Il12a* KO *n* = 7 mice). **b** The representative plots show T-bet and CXCR3 expression in MP CD4^+^ T cells from each group while the bar graph indicates the frequency (mean ± SD) of T-bet^high^CXCR3^+^ fraction among the same CD4^+^ T cell population (WT *n* = 8 mice; *Il12b* KO *n* = 8 mice; *Il12a* KO *n* = 7 mice). Data are pooled from three independent experiments performed. (**c**) IL-23A p19 is dispensable for T-bet^high^ MP differentiation. The bar graph shows the frequency (mean ± SD) of T-bet^high^CXCR3^+^ cells among MP cells from WT and *Il23a* KO mice (*n* = 5 mice). Data are representative of two independent experiments performed. (**d** and **e**) DC1 tonically produce IL-12B p40. The representative contour plots in (**d**) show p40 expression by the indicated DC subsets from WT animals while the histograms in (**e**) depict YFP levels expressed by the same DC subsets from p40-YFP reporter mice. The filled histograms show negative control staining using non-reporter mice. The bar graphs indicate the p40^+^ and YFP^+^ fractions (mean ± SD) among indicated cell populations (*n* = 5 mice). Data are representative of three independent experiments. (**f** and **g**) Activated DC1 produce tonic IL-12B p40. The representative plots in (**f**) depict p40 expression in MHCII^very high^CD86^high^CD40^high^ and MHCII^high^CD86^low^CD40^low^CD8α^+^ DCs from WT mice while the histograms in (**g**) show YFP expression in the same DC subsets from p40-YFP reporter mice. The bar graphs in (**f**) and (**g**) show the p40^+^ and YFP^+^ fractions (mean ± SD), respectively, among indicated CD8α^+^ DC subpopulations (*n* = 3 mice). Data are representative of two independent experiments performed. (**h** and **i**) p40^+^ DC1 are localized in the splenic T-cell zone. Different cell types in spleen of p40-YFP reporter mice were analyzed by multicolor tissue imaging. B220: yellow; CD4: purple; CD8: red; CD11c: blue; YFP: green. Scale bars show (**h**) 150 μm and (**i**) 15 μm. Images are representative of four sections from two mice. A two-sided *t*-test was performed to assess statistical significance. Source data are provided as a Source Data file.

In the case of conventional Ag-specific effector T cells, naïve CD4^+^ T lymphocytes differentiate into a T-bet^+^ Th1 subset in the presence of IL-12, a cytokine composed of IL-12B p40 and IL-12A p35 subunits^13,17–19^. To test whether IL-12 is also required to drive MP differentiation toward the T-bet^high^ subset in steady state, we first compared MP cell levels in wild-type (WT), *Il12b* KO, and *Il12a* KO mice. While the frequency of total MP cells among CD4^+^ T cells was unchanged, the T-bet^high^ MP cell fraction was significantly reduced in both KO mice (Fig. 2a, b). In contrast, deficiency in *Il23a*, encoding the IL-23A p19 chain which together with IL-12B p40 forms the IL-23 heterodimer, did not affect T-bet^high^ MP development (Fig. 2c). This data argued that homeostatically produced IL-12 but not IL-23 is essential for optimal T-bet^high^ MP differentiation while dispensable for MP generation itself.

To determine which cells serve as the source of the IL-12 responsible for maintaining a tonic level of T-bet^high^ MP cells, we measured IL-12B p40 expression at steady state in various cell populations from WT and control *Il12b*-deficient mice. Interestingly, CD8α^+^ DC1 were found to selectively produce IL-12B p40 under homeostatic conditions in the former animals (Fig. 2d and Supplementary Fig. 2a; gating strategy is described in Methods). Similarly, in IL-12B p40-YFP mice that report *Il12b* promoter activity, DC1 expressed the highest levels of YFP (Fig. 2e) and the YFP^+^ fraction was confirmed to selectively express p40 protein (Supplementary Fig. 2b). Moreover, DC1 expressed the highest levels of IL-12A p35 among the DC subsets examined and all p40^+^ DC1 doubly stained for p35 (Supplementary Fig. 2c, d). In additional experiments, we compared the expression of the p40 and p70 IL-12 proteins by ELISA in culture supernatants of purified DC1. Although p40 production was readily measured, p70 levels were below the limit of detection (Supplementary Fig. 3) consistent with other studies attempting to measure small amounts of the heterodimer^26,27^. These results identify DC1 as the primary source of homeostatic IL-12 p40, p35, and bioactive IL-12.

DCs can retain various activation states^28^. To determine the relationship between activation status of DCs and their IL-12 production in homeostasis, we assayed expression levels of various activation markers of DC1 together with their IL-12 production. To do so we measured IL-12B p40 because DC1 are essentially all IL-12A p35^+^ while p40 is expressed only by a small fraction of these cells (Supplementary Fig. 2d). As demonstrated in Fig. 2f, activated DCs (i.e., MHCII^very high^CD86^high^CD40^high^; gating strategy shown in Methods) expressed higher levels of p40 than did the remaining populations, an observation that was also confirmed using p40-YFP mice (Fig. 2g). This observation suggests that the activation status of DCs determines the amount of IL-12 produced in steady state as it does during infectious challenge^22^.

To examine the spatial distribution of IL-12-producing DC1 in vivo at baseline, we performed multiplex immunofluorescent confocal imaging analysis using p40-YFP reporter mice. YFP^+^ cells were localized in the T-cell zone and co-stained for both CD11c and CD8 (Fig. 2h, i). These observations indicate that IL-12-producing CD8α^+^ DCs are present at an anatomical site where they can closely interact with CD4^+^ T cells in steady state.

**Fig. 3:**
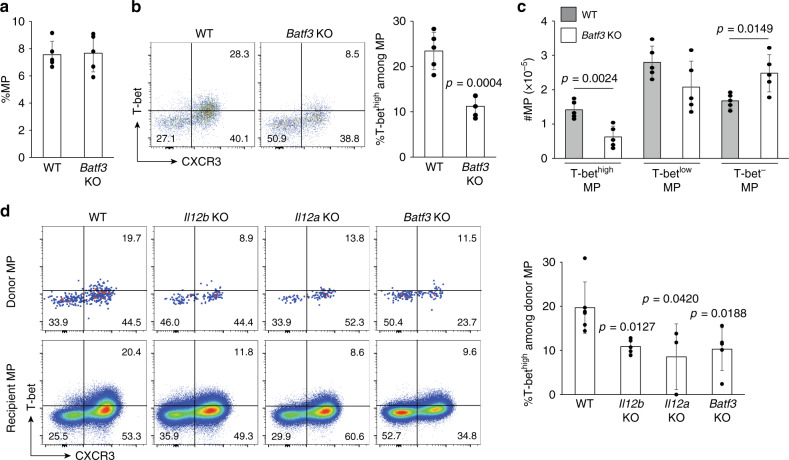
DC1 are essential for optimal T-bet^high^ MP cell differentiation. (**a**–**c**) BatF3-sensitive DCs are essential for the optimal differentiation of MP cells. **a** The bar graph shows the frequency (mean ± SD) of MP cells among CD4^+^ T lymphocytes from WT and *Batf3* KO mice (*n* = 5 mice). **b** The representative dot plots depict expression of T-bet and CXCR3 in MP CD4^+^ T cells from each group while the bar graph indicates the frequency (mean ± SD) of the T-bet^high^CXCR3^+^ subpopulation among the same MP population (*n* = 5 mice). **c** Total cell numbers (mean ± SD) of each of the MP subsets in (**b**) (*n* = 5 mice). Data are representative of two independent experiments. (**d**) CD4^+^ T cell-extrinsic BatF3 and IL-12 support differentiation of naïve CD4^+^ T lymphocytes toward the T-bet^high^ MP subset. Sorted naïve CD4^+^ T cells were transferred into congenic WT and the indicated KO mice and analyzed 3 weeks later. The representative dot plots depict T-bet and CXCR3 expression by donor- and recipient-derived MP cells while the bar graph shows the frequency (mean ± SD) of T-bet^high^CXCR3^+^ subpopulation among MP donor cells (WT *n* = 6 mice; *Il12b* KO *n* = 5 mice; *Il12a* KO *n* = 3 mice; *Batf3* KO *n* = 5 mice). Data are pooled from two independent experiments performed. A two-sided *t*-test was performed to assess statistical significance. Source data are provided as a Source Data file.

We next asked whether DC1 are essential for T-bet^high^ MP differentiation. To do so we compared MP cell levels in WT with *Batf3* KO mice that lack the DC1 subset^29^. While the frequency of MP cells was unaltered in *Batf3* KO animals (Fig. 3a), their expression of T-bet was significantly reduced (Fig. 3b). Moreover, the absolute number of T-bet^high^ MP cells was lower while that of T-bet^−^ cells higher in the same KO mice (Fig. 3c). To confirm that the differentiation of T-bet^high^ MP cells depends on T cell-extrinsic BatF3 and IL-12, naïve CD4^+^ T cells were transferred into congenic WT and *Batf3*, *Il12b*, and *Il12a* KO mice and the donor cells analyzed 3 weeks later. Among the donor-derived MP cells generated, the T-bet^high^ fraction was significantly lowered in the absence of recipient-derived BatF3 or either IL-12 subunit (Fig. 3d). Together these findings support the hypothesis that in steady state, DC1 producing IL-12 promote the differentiation of the T-bet^high^ MP cell subpopulation.

DC1 have been previously reported to affect immune responses in multiple types of lymphocytes^30^. To test if this is also the case in steady state, we analyzed CD8^+^ T- and NK-cell populations in WT and *Batf3* KO mice. Both the frequency of CD8^+^ T lymphocytes with a memory-phenotype and their T-bet expression were unchanged in KO mice (Supplementary Fig. 4a, b). Similarly, both the number of NK cells and their T-bet expression were intact (Supplementary Fig. 4c, d) in the *Batf3*-deficient animals. These findings argue that in noninflammatory conditions, DC1 mainly affect T-bet expression levels in MP CD4^+^ T but not in MP CD8^+^ T or NK lymphocytes.

### IL-12 is dispensable for maintenance of T-bet^high^ MP cells

**Fig. 4:**
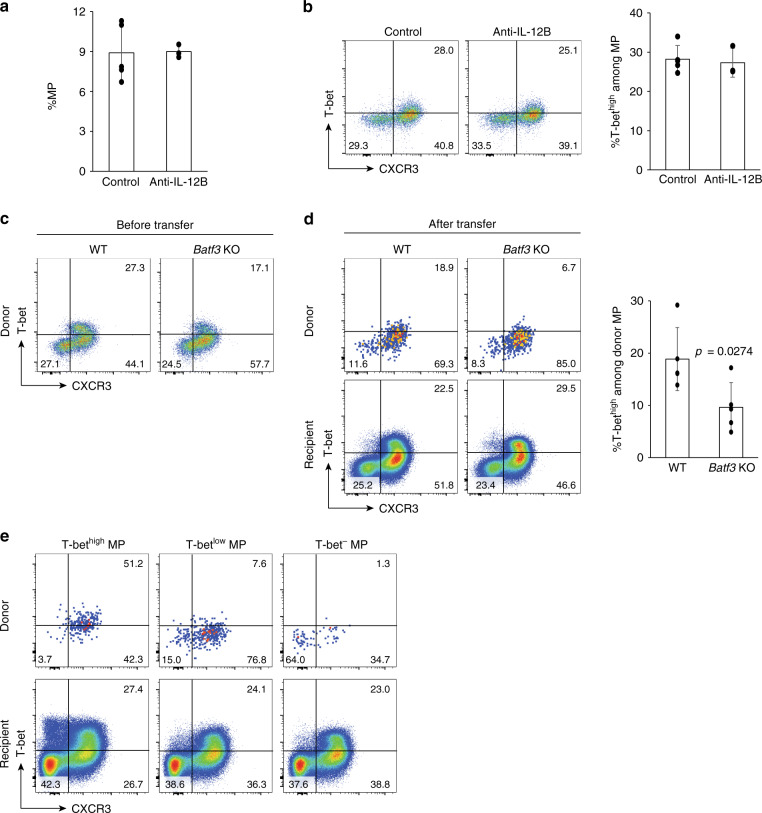
Stability of mature T-bet^high^, T-bet^low^, and T-bet^−^ MP cells and their IL-12-independence. (**a** and **b**) Blockade of IL-12B p40 does not hamper the T-bet^high^ MP subpopulation. WT mice received anti-IL-12B p40 mAb or control IgG every other day for 1 week and were analyzed for MP CD4^+^ T cells. **a** The bar graph shows the frequency (mean ± SD) of MP cells among CD4^+^ T lymphocytes (Control *n* = 5 mice; Anti-IL-12B *n* = 3 mice). **b** The representative dot plots display T-bet and CXCR3 expression in MP cells while the bar graph indicates the frequency (mean ± SD) of T-bet^high^ subpopulation among MP cells from each group (Control *n* = 5 mice; Anti-IL-12B *n* = 3 mice). (**c** and **d**) *Batf3* KO MP cells have lower levels of T-bet^high^ subpopulation before and after transfer into congenic WT mice. Sorted MP cells from CD45.2^+^ WT and *Batf3* KO mice were transferred into CD45.1^+^ WT recipients and the donor as well as recipient MP cells analyzed 10 days later. The representative dot plots show T-bet and CXCR3 expression in donor MP cells (**c**) before and (**d**) after transfer while the bar graph indicates the frequency (mean ± SD) of the T-bet^high^ subpopulation among transferred MP cells (*n* = 5 mice). (**e**) MP cells largely maintain their T-bet expression levels after generation. T-bet^high^, T-bet^low^, or T-bet^−^ MP cells were sorted from CD45.2^+^ T-bet-ZsGreen reporter mice, transferred into CD45.1^+^ WT recipient animals, and analyzed for their T-bet and CXCR3 expression 10 days later. Representative dot plots display T-bet and CXCR3 expression by donor as well as recipient MP cells from three independent host mice per group. A two-sided *t*-test was performed to assess statistical significance. Source data are provided as a Source Data file.

The above observation that IL-12 promotes T-bet^high^ MP differentiation from naïve precursors (Fig. 3d) prompted us to test whether the same cytokine also contributes to the maintenance of the T-bet^high^ subset after its generation. To address this question, we used a previously described method in which MP cell maintenance as opposed to generation can be assessed^2^. In this procedure, WT mice are treated for 1 week with a strongly neutralizing anti-IL-12B p40 mAb previously shown to nearly completely block IL-12 signaling^2^ and then assayed for T-bet^high^ MP cell levels. Since within 1 week new MP generation is known to be minimal^2^, any MP cells detected after anti-IL-12B administration should represent those present before treatment. When this experiment was performed, anti-IL-12B treatment did not alter the frequency of either the total MP cells or the T-bet^high^ subpopulation (Fig. 4a, b), consistent with the hypothesis that T-bet^high^ MP cells become less dependent on IL-12 signaling for their maintenance after generation from naïve precursors.

To assess the possibility that T-bet^high^ MP cells may derive homeostatically from T-bet^−^ or T-bet^low^ subsets, MP cells obtained from naïve WT or *Batf3* KO mice were transferred into CD45.1^+^ WT recipients and analyzed for their T-bet and CXCR3 expression 10 days later. As expected (Fig. 3b), the T-bet^high^ fraction was reduced in the donor *Batf3* KO but not the WT MP cell population (Fig. 4c), and this lower expression of T-bet was maintained after transfer although total T-bet expression was downregulated in both groups (Fig. 4d). To further investigate the stability of MP subpopulations in steady state, T-bet^high^, T-bet^low^, or T-bet^−^ MP cells were sorted from CD45.2^+^ WT T-bet-ZsGreen reporter mice and transferred individually into CD45.1^+^ WT recipient animals. When analyzed 10 days following transfer, all three MP subsets maintained their relative T-bet expression levels while in the case of the T-bet^high^ and T-bet^low^ cells downregulating the transcription factor in a minor fraction of the total population and conversely upregulating expression on a small population of formerly T-bet^−^ cells (Fig. 4e). Together these data argue that T-bet^high^ MP cells are mainly generated from naïve precursors and not from T-bet^−^ or T-bet^low^ MP subsets while providing evidence for a minor degree of plasticity between the three MP subpopulations in steady state.

### Tonic IL-12 is induced by TLR-MyD88 but not IFN-γ signaling

Because IL-12 is induced by LPS as well as other microbial-derived products and further upregulated by CD40, and in some cases, IFN-γ^21,22,31,32^, we next sought to determine the role of these three alternative stimuli in steady state. Initial experiments indicated that while MP and NK lymphocyte populations express IFN-γ under homeostatic conditions in IFN-γ-YFP reporter mice (Supplementary Fig. 5a), this IFN-γ is dispensable for tonic IL-12 expression and MP differentiation since WT and *Ifng* KO mice had similar levels of p40^+^ DC1 as well as T-bet^high^ MP cells (Supplementary Fig. 5b, c).

**Fig. 5:**
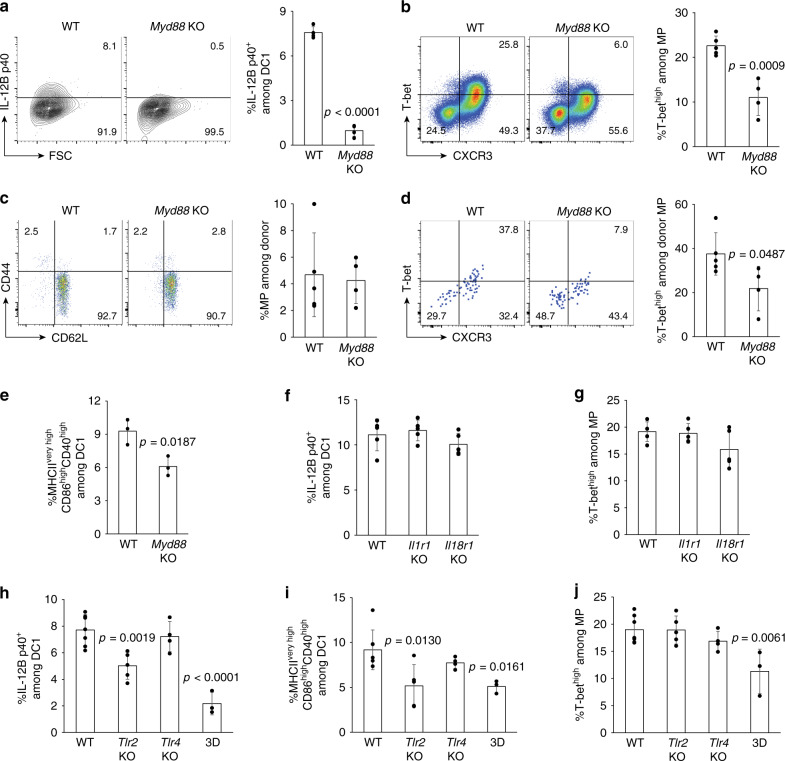
TLR-MyD88 signaling homeostatically induces IL-12 and supports MP differentiation. (**a** and **b**) MyD88 is essential for homeostatic IL-12B p40 production and optimal T-bet^high^ MP differentiation. **a** Representative contour plots indicating p40 expression in CD8α^+^ DCs from WT and *Myd88* KO animals and a bar graph showing the fraction (mean ± SD) of p40^+^ among CD8α^+^ DCs from each group (*n* = 4 mice). **b** The dot plots show T-bet and CXCR3 expression in MP CD4^+^ T lymphocytes from each group while the bar graph indicates the frequency (mean ± SD) of T-bet^high^CXCR3^+^ cells among the total MP population (WT *n* = 5 mice; *Myd88* KO *n* = 4 mice). Data are representative of two independent experiments performed. (**c** and **d**) T cell-extrinsic MyD88 supports T-bet^high^ MP differentiation from naïve precursors. Sorted naïve CD4^+^ T lymphocytes were transferred into congenic WT and *Myd88* KO animals and analyzed 3 weeks later. The representative dot plots indicate expression of (**c**) CD44 and CD62L in the donor cell population and (**d**) T-bet and CXCR3 in MP donor cells from each group. The bar graphs show the frequency (mean ± SD) of (**c**) MP cells among donor cell population and (**d**) T-bet^high^CXCR3^+^ subset among MP donor cells (WT *n* = 5 mice; *Myd88* KO *n* = 4 mice). Data are pooled from two independent experiments. (**e**) MyD88 tonically activates DC1. The bar graph indicates the frequency (mean ± SD) of MHCII^very high^CD86^high^CD40^high^ cells among CD8α^+^ DCs (*n* = 3 mice). (**f** and **g**) IL-1/18 signaling is dispensable for tonic IL-12B p40 production and T-bet^high^ MP differentiation. The bar graphs show the frequency (mean ± SD) of (**f**) p40^+^ cells among CD8α^+^ DCs and (**g**) T-bet^high^CXCR3^+^ cells within MP cells from the indicated KO animals (*n* = 5 mice). (**h**–**j**) Contribution of multiple TLRs to tonic IL-12B p40 production and MP differentiation. The bar graphs indicate the fraction (mean ± SD) of (**h**) p40^+^ cells among CD8α^+^ DCs, (**i**) MHCII^very high^CD86^high^CD40^high^ cells among CD8α^+^ DCs, and (**j**) T-bet^high^CXCR3^+^ cells among MP lymphocytes obtained from indicated animals (WT *n* = 7 mice; *Tlr2* KO *n* = 5 mice; *Tlr4* KO *n* = 4 mice; 3D *n* = 3 mice). Data are pooled from two independent experiments. A two-sided *t*-test was performed to assess statistical significance. Source data are provided as a Source Data file.

We next analyzed the role for microbial- and other-derived agonists in IL-12B p40 expression and T-bet^high^ MP differentiation. To do so we used mice deficient in MyD88, an adapter molecule which plays a prominent role in TLR and IL-1R signaling^33^. Expression of p40 by DC1 was largely abolished in the absence of MyD88 (Fig. 5a), and in parallel, T-bet^high^ MP differentiation was partially and significantly inhibited (Fig. 5b). To examine if T cell-extrinsic MyD88 affects MP differentiation, naïve CD4^+^ T cells from WT donors were transferred into congenic WT and *Myd88* KO recipient animals and donor cells analyzed 3 weeks later. While MP generation from naïve cells was unaffected in *Myd88* KO hosts, T-bet^high^ MP differentiation was significantly reduced (Fig. 5c, d), confirming the importance of T cell-extrinsic MyD88 signaling in the latter process. To examine the activation status of DC1 in *Myd88* KO mice, we determined the fraction of DCs with an activated phenotype among CD8α^+^ DCs. The frequency of activated DC1 was reduced in *Myd88* KO mice (Fig. 5e). Taken together, these findings show that T cell-extrinsic MyD88 signaling activates DC1 to prime tonic IL-12 expression and that this process enhances differentiation of MP cells toward the T-bet^high^ subpopulation.

To determine which pathway, TLR or IL-1/18 receptor signaling, contributes to the above MyD88-dependent responses, we analyzed a series of mouse strains deficient in components of these pathways. While p40 production by DC1 and T-bet^high^ MP cell differentiation were intact in *Il1r1* and *Il18r1* KO mice (Fig. 5f, g), DC1 p40 and activation marker expression (MHCII, CD86, and CD40) as well as T-bet^high^ MP differentiation responses were significantly impaired in 3D mice mutated in *Unc93b1* that encodes a protein required for TLR3/7/9/11/12/13 trafficking and function^34–38^ (Fig. 5h–j). In contrast, no effect on these parameters was evident in *Tlr4* KO animals, arguing against a role for commensal LPS, while an intermediate loss in DC function was seen in *Tlr2* KO mice (Fig. 5h–j). Similarly, partial effects on steady-state p40 production was seen in DC1 from *Tlr3*, *7*, *9* and *11/12* KO mice (Supplementary Fig. 6). Together these observations suggest that the effect of MyD88 deficiency on DC p40 synthesis reflects a role for multiple TLRs but not IL-1/18 or TLR4 signaling.

### Tonic IL-12 is maintained by CD40L on CD4^+^ T cells

**Fig. 6:**
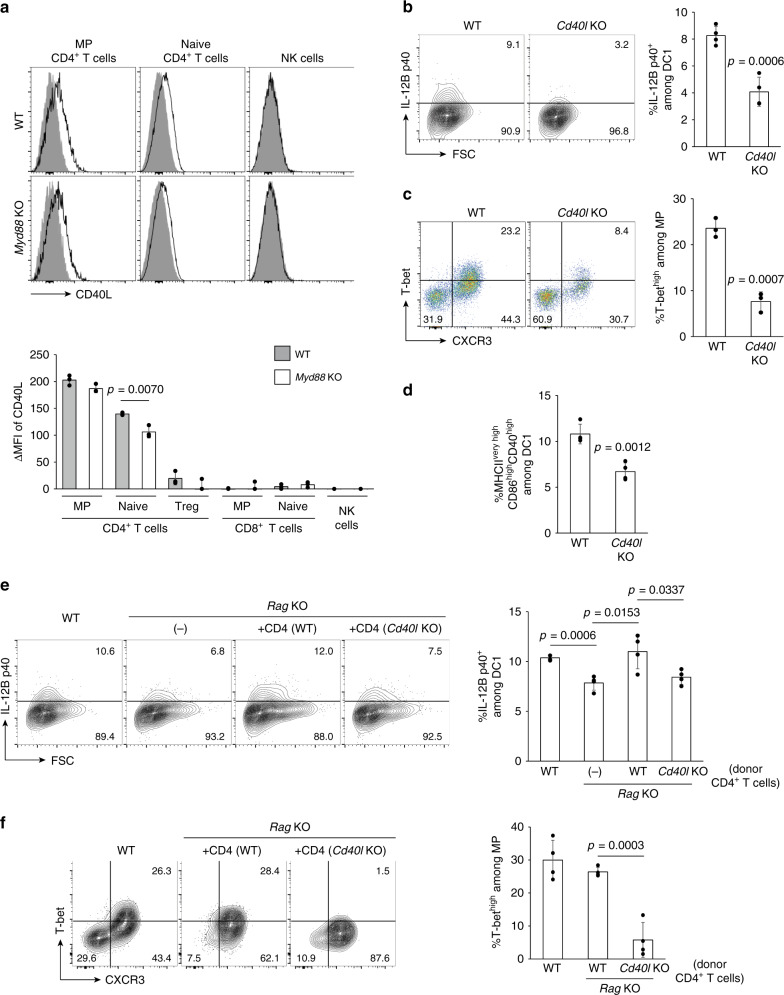
CD40L on CD4^+^ T lymphocytes promotes tonic IL-12 expression and MP differentiation. (**a**) CD4^+^ T cells express CD40L independently of MyD88 signaling. The open histograms depict CD40L expression by various subpopulations of lymphoid cells from indicated animals while the filled histograms show negative control staining. The bar graph depicts the ΔMFI (mean ± SD) of CD40L expression in the indicated cell populations from each group (*n* = 3 mice). Data shown are representative of two independent experiments performed. (**b** and **c**) CD40L enhances DC1 IL-12B p40 expression and T-bet^high^ MP differentiation. **b** The representative contour plots show p40 expression by CD8α^+^ DCs obtained from WT and *Cd40l* KO animals while the bar graph indicates the p40^+^ fraction (mean ± SD) among CD8α^+^ DCs from each group (*n* = 4 mice). **c** Dot plots displaying T-bet and CXCR3 expression in MP CD4^+^ T cells from each group with a bar graph showing the frequency (mean ± SD) of T-bet^high^CXCR3^+^ cells among the same T-cell subpopulation (*n* = 3 mice). Data shown are representative of two independent experiments. (**d**) CD40L tonically activates DC1. The bar graph indicates the frequency (mean ± SD) of MHCII^very high^CD86^high^CD40^high^ cells among CD8α^+^ DCs (*n* = 4 mice). Data are representative of two independent experiments performed. (**e** and **f**) CD4^+^ T lymphocytes enhance IL-12B p40 expression in DC1 via CD40L to promote their own differentiation. CD4^+^ T lymphocytes sorted from WT or *Cd40l* KO mice were transferred into *Rag* KO animals, and donor and recipient cell populations examined 3 weeks later. WT and untransferred *Rag* KO animals were also analyzed. **e** The representative contour plots depict p40 expression in CD8α^+^ DCs of the hosts from indicated groups while the bar graph shows the frequency (mean ± SD) of p40^+^ cells among CD8α^+^ DCs from each group. **f** Contour plots displaying T-bet and CXCR3 expression in CD44^high^CD62L^low^CD4^+^ T cells from the indicated groups and a bar graph indicating the frequency of T-bet^high^CXCR3^+^ cells (mean ± SD) among the same cell subpopulations (*n* = 4 mice). Data are representative of two independent experiments performed. A two-sided *t*-test was performed to assess statistical significance. Source data are provided as a Source Data file.

CD40-CD40L signaling has been previously shown to enhance IL-12 production by microbially stimulated DCs^22^. For this reason we next assessed the effect of CD40 signaling on IL-12 expression in steady state. Initial experiments revealed that T-bet^high^, T-bet^low^, and T-bet^−^ MP CD4^+^ T lymphocytes, and to a lesser extent, naïve CD4^+^ T cells, expressed CD40L under homeostatic conditions while CD8^+^ T and NK cell populations did not (Fig. 6a and Supplementary Fig. 7). The observed tonic CD40L expression was largely MyD88-independent (Fig. 6a).

To examine if CD40L expression enhances DC1 IL-12 production as well as T-bet^high^ MP differentiation, we compared these parameters in WT versus *Cd40l* KO animals. Tonic p40 expression was significantly reduced and T-bet^high^ MP differentiation inhibited in the KO mice (Fig. 6b, c). Consistent with this observation, DC1 were less activated in *Cd40l* KO than in WT mice (Fig. 6d). These results demonstrate that CD40-CD40L signaling promotes IL-12 production in DC1 by activating this DC subset thereby enhancing MP differentiation in steady state.

The above findings raised the question of whether CD40L expressed on CD4^+^ T cells is crucial for optimal tonic IL-12 expression in DC1. To address this possibility, we adoptively transferred CD45.2^+^CD4^+^ WT T cells to CD45.1^+^ *Rag* KO recipients. We found that several weeks following transfer, a majority of the donor lymphocytes had converted into MP cells (Supplementary Fig. 8a) consistent with our previous finding^2^. At their plateau, the equilibrated MP cells contained T-bet^high^ and CD40L^+^ subpopulations (Supplementary Fig. 8b, c), confirming that the MP cells established by this method closely resemble those present in normal, unmanipulated animals in terms of their differentiation status. We then transferred CD4^+^ T cells from WT or *Cd40l* KO animals into *Rag* KO mice and measured IL-12B p40 expression levels in DC1 of the host 3 weeks later. *Rag* KO mice that had not received donor cells expressed lower amounts of IL-12B p40 compared with WT controls, and CD4^+^ T cell transfer restored expression of the cytokine to WT levels (Fig. 6e). Similar results were obtained when p40-YFP reporter *Rag* KO animals were used as the recipient (Supplementary Fig. 8d). In contrast, donor cells obtained from *Cd40l* KO animals failed to rescue p40 expression (Fig. 6e), and consistent with this, WT but not *Cd40l* KO donor cells differentiated into T-bet^high^ MP cells (Fig. 6f). Taken together, these results argue that CD40L expressed on CD4^+^ T lymphocytes plays a major role in maintaining tonic IL-12 expression in DC1.

### MP differentiation correlates with TCR affinity to self Ags

**Fig. 7:**
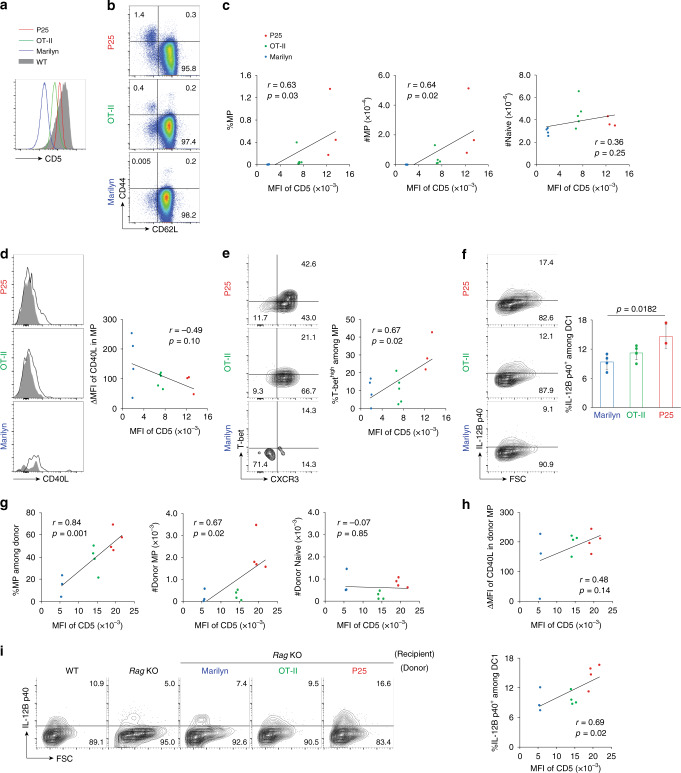
CD5 levels on CD4^+^ T lymphocytes correlate with their capacity to upregulate p40 and differentiate into T-bet^high^ cells. (**a**–**f**) CD5 levels on TCR-Tg CD4^+^ T lymphocytes are positively associated with the extent of MP differentiation and DC1 p40 expression but not with CD40L expression or naïve cell levels. **a** CD5 levels on CD4^+^ T cells from P25, OT-II, and Marilyn TCR-Tg and WT mice. **b** CD44 and CD62L expression in CD4^+^ T lymphocytes from indicated animals. **c** MFI of CD5 expression obtained in (**a**) versus the frequency and the number of MP as well as naïve cells among CD4^+^ T cells obtained in (**b**) plotted against each other. **d** The representative histograms show CD40L expression on MP lymphocytes from each TCR-Tg animal while the scatter plot indicates the relationship between ΔMFI of CD40L and MFI of CD5. **e** The contour plots depict expression of T-bet and CXCR3 in MP cells from each TCR-Tg animal and the scatter plot displays the relationship between the frequency of T-bet^high^CXCR3^+^ cells among the MP population and the MFI of CD5. **f** The contour plots display p40 expression in CD8α^+^ DCs from the indicated animals while the bar graph shows the frequency (mean ± SD) of p40^+^ cells among the same DC subset. Data are pooled from two independent experiments (Marilyn *n* = 4 mice; OT-II *n* = 5 mice; P25 *n* = 3 mice). (**g**–**i**) CD5 levels in CD4^+^ T lymphocytes positively correlate with their capacity to augment DC1 p40 expression. Naïve CD4^+^ T lymphocytes sorted from P25, OT-II, and Marilyn TCR-Tg mice were transferred into *Rag* KO animals and donor-derived CD4^+^ T cells and host-derived CD8α^+^ DCs analyzed 3 weeks later. **g** and **h** MFI of CD5 plotted against (**g**) the frequency and the number of CD44^high^CD62L^low^ as well as CD44^low^CD62L^high^ donor cells and (**h**) ΔMFI of CD40L in the different transgenic T-cell populations following adaptive transfer. **i** Representative contour plots showing p40 expression in host-derived CD8α^+^ DCs from the indicated groups together with a scatter plot depicting the relationship between MFI of CD5 and the frequency of p40^+^CD8α^+^ DCs from each group. Results from the analysis of untransferred WT and *Rag* KO mice are also included. Data are pooled from two independent experiments (Marilyn *n* = 3 mice; OT-II *n* = 4 mice; P25 *n* = 4 mice). Red, green, and blue lines/dots show P25, OT-II, and Marilyn TCR-Tg T cells, respectively. A two-sided *t*-test was performed to assess significance. r, Pearson’s correlation coefficient. Source data are provided as a Source Data file.

The above results implicated CD40L expression by CD4^+^ T lymphocytes as a key contributor to the maintenance of IL-12 expression by DC1 in steady state. Because homeostasis of naïve and MP CD4^+^ T lymphocytes is governed by a mechanism dependent on recognition of available Ags including self^1,2^, we hypothesized that TCR affinity to self determines the capacity of CD4^+^ T lymphocytes to maintain IL-12 levels in DC1 and thus their own differentiation toward the T-bet^high^ MP cells. To examine the relationship between TCR affinity of CD4^+^ T lymphocytes to self Ags and their capacity to upregulate DC1 IL-12 expression and trigger T-bet^high^ MP differentiation, we utilized three TCR-transgenic (Tg) mouse lines, P25, OT-II, and Marilyn. CD4^+^ T cells from naïve animals of these three lines express different levels of CD5 (Fig. 7a), which is known to be a reliable marker that reflects strength of TCR affinity to self^39^. When these CD4^+^ T lymphocytes were assayed for their MP and naïve cell frequencies, a positive correlation between expression levels of CD5 and the number of MP but not naïve cells in steady state was observed (Fig. 7b, c), consistent with the notion that MP generation is dependent on self-recognition. While there was no significant correlation between the CD5 and CD40L expression in the MP cells from the three lines (Fig. 7d), CD5 levels positively correlated with their T-bet levels (Fig. 7e) and in parallel, with IL-12B p40 produced by DC1 (Fig. 7f). Taken together, these results suggest that CD4^+^ T lymphocytes with higher TCR affinity to self can generate more MP cells and promote their differentiation into the T-bet^high^ subset via upregulation of IL-12. The data also argue that MP cells can enhance IL-12 expression by DC1 but do not rule out a role for their naïve CD4^+^ T-cell counterparts in performing the same function.

The observations that MP cells can sustain DC IL-12B levels and that CD40L per-cell levels on MP cells are the same among P25, OT-II, and Marilyn TCR-Tg mice suggest that TCR affinity to self determines the number of MP cells generated from naïve precursors, which eventually controls the amount of IL-12 expressed by DC1. To test this hypothesis we transferred naïve CD4^+^ T cells from the 3 TCR-Tg lines into *Rag* KO mice. When analyzed 3 weeks later, CD5 expression levels on donor cells correlated with the frequency and the number of MP cells generated but not with their CD40L levels or the number of naïve CD4^+^ T cells (Fig. 7g, h) thus confirming the data obtained in steady state in intact mice (Fig. 7a–d). While the deficiency in T and B lymphocytes in *Rag* KO mice resulted in decreased p40 production by DC1, TCR-Tg T-cell transfer restored its expression in varying degrees. Importantly, there was a significant correlation between TCR affinity of the donor cells to self and their capacity to upregulate p40 expression in CD8α^+^ DCs of the recipients (Fig. 7i). Together these findings argue that TCR affinity to self Ags regulates the number of MP cells generated and indirectly IL-12 expression by DC1.

### Foreign agonists are dispensable for tonic IL-12 production

**Fig. 8:**
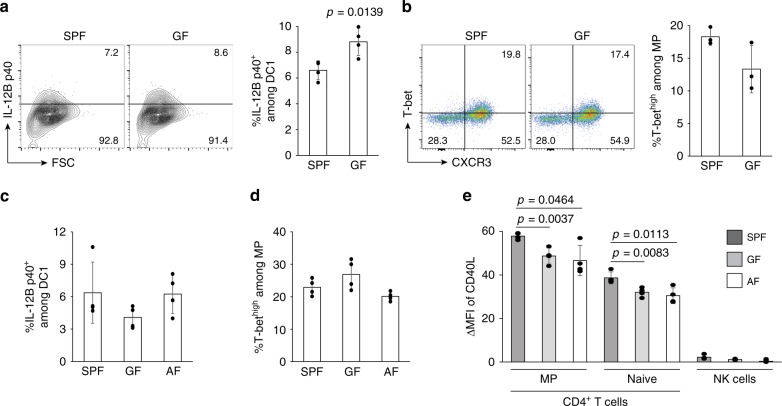
Microbial products are dispensable for tonic p40 expression and T-bet^high^ MP differentiation. (**a** and **b**) Commensal bacteria are not required for IL-12B p40 expression or MP differentiation. **a** Representative contour plots comparing p40 expression in splenic CD8α^+^ DCs from SPF and GF mice together with a bar graph showing the frequency of p40^+^ cells (mean ± SD) among CD8α^+^ DCs from each group (*n* = 4 mice). **b** Representative dot plots depicting T-bet and CXCR3 expression in MP cells together with a summary graph indicating the frequency (mean ± SD) of T-bet^high^CXCR3^+^ lymphocytes among the same CD4^+^ T-cell subpopulations from each group (*n* = 3 mice). Data are representative of two independent experiments performed. (**c**–**e**) AF mice display homeostatic IL-12B p40 expression and MP differentiation indistinguishable from both SPF and GF mice. The bar graphs show the frequency (mean ± SD) of (**c**) p40^+^ cells among CD8α^+^ DCs and (**d**) T-bet^high^CXCR3^+^ cells among MP CD4^+^ T lymphocytes as well as (**e**) ΔMFI of CD40L expression in the indicated cell populations from SPF, GF, and AF mice (*n* = 4 mice). A two-sided *t*-test was performed to assess statistical significance. Source data are provided as a Source Data file.

The results presented above demonstrated that multiple TLR signals are critical for both optimal IL-12B p40 production by DC1 and T-bet^high^ MP differentiation (Fig. 5 and Supplementary Fig. 6). They further showed that TCR affinity of CD4^+^ T lymphocytes to self Ags correlates with the extent of MP cell development and that MP cells themselves can promote DC cytokine expression (Figs. 6 and 7). Because of this evidence implicating self-recognition, we asked whether stimulation by foreign-derived agonists is necessary for p40 expression and T-bet^high^ MP differentiation or whether stimulation by self-components is sufficient. To do so, we compared these parameters in GF versus SPF mice. Both IL-12B p40 expression by DC1 and T-bet^high^ MP levels were equivalent in the two groups of mice (Fig. 8a, b), arguing that commensal-derived components are not required for either IL-12B p40 expression or T-bet^high^ MP differentiation.

To rule out the possible role of microbial-derived components and foreign Ags contained in food, AF animals that are simultaneously GF and deprived of virtually all foreign-derived products and Ags in their food^6^ were analyzed for IL-12B p40 expression and T-bet^high^ MP differentiation as well as CD40L expression. These AF animals exhibited unaltered p40 production by CD8α^+^ DCs and T-bet^high^ MP cell differentiation (Fig. 8c, d) and only a minor decrease in CD40L expression in MP and naïve CD4^+^ T lymphocytes relative to SPF mice (Fig. 8e). Together, the above experiments argue that commensal- or food-derived agonists are dispensable for tonic IL-12 expression in DC1 and T-bet^high^ MP differentiation, pointing to a possible contribution of self-derived components in stimulating these responses.

## Discussion

In the present study, we have characterized T-bet^high^ MP lymphocytes as a cell population equipped with innate Th1-like effector function and addressed the mechanisms underlying their differentiation. We found that in steady state, MP cells differentiate into a T-bet^high^ subset in the presence of IL-12 derived from DC1 endowed with an activated phenotype. This homeostatic IL-12 production depends on TLR-MyD88 signaling and is further enhanced by DC interaction with CD40L expressed by CD4^+^ T lymphocytes. Our findings further suggest that self-derived agonists are sufficient for this tonic IL-12 expression. Together, our observations reveal IL-12 as a homeostatic cytokine required for the optimal differentiation of T-bet^high^ MP T lymphocytes.

As noted above, CD4^+^ MP cells are distinct from conventional memory cells in their requirements for development and maintenance as well as their function^1,2,4,8^. Nevertheless, in contrast to conventional memory cells, there has been a dearth of information concerning the functional heterogeneity of CD4^+^ MP cells. In this report, we formally show that MP cells consist of highly differentiated T-bet^high^ and less activated T-bet^low/−^ subpopulations, with innate Th1-like effector function almost exclusively associated with the former subpopulation. This observation suggests that in analogy with conventional memory cells CD4^+^ MP cells may be composed of functionally unique subsets. Consistent with this hypothesis, we found that some T-bet^−^ cells tonically express the RORγt transcription factor required for Th17 differentiation and function. This RORγt^+^ population is small under homeostatic conditions (Fig. 1b). We propose however, that it may expand in situations where tonic Th1-like differentiation is hampered such as in *Batf3* KO mice and are currently exploring this hypothesis. Although we have failed to detect an additional Gata3^high^ MP fraction in steady state, it remains to be seen whether such a population would emerge in response to appropriate immunologic stimulation.

In addition to the above RORγt^+^ fraction, some T-bet^−^ MP cells may be derived from the T-bet^high^ subpopulation since following adoptive transfer some T-bet^high^ cells gradually converted to T-bet^low^ and because T-bet^low^ and T-bet^−^ cells were interchangeable (Fig. 4e). Therefore, it is possible that the T-bet^−^ fraction consists of a mixture of subsets of different lineage as well as less activated former T-bet^high^ cells.

In the present study we provide evidence showing that generation of T-bet^high^ MP cells is significantly enhanced by both IL-12 p40 and p35 (presumably reflecting IL-12 p70) in steady state. While IL-12 is well established as an inflammatory cytokine essential for the induction of Th1-type effector and memory T lymphocytes^17–20^, our observations reveal an additional homeostatic function for this cytokine in T-bet^high^ MP cell differentiation. IL-12B p40 is known to be constitutively expressed by CD8α^−^CD11b^−^ DCs in the small intestine and CD103^+^ DCs in mesenteric lymph nodes, the former population producing this cytokine in response to commensal microbiota^40,41^. In addition, low level steady-state IL-12B expression by certain populations of DCs in the spleen and cutaneous lymph nodes has been described in prior studies^30,42^. Although these previous findings suggested homeostatic roles for IL-12, the functional significance of these observations was not fully elucidated except in the case of one study in which constitutive IL-12 production by migratory CD103^+^ DCs was shown to antagonize type 2 immunity in helminth infection^41^. The work presented here reveals that constitutive IL-12 is also essential for the optimal generation of innate-like T-bet^high^ MP cells in steady state. Although tonic IL-12 p70 expression by purified DC1 was below the detection limit by ELISA, it is possible that the heterodimer may nevertheless be present in a form bound to viable DCs and act on T lymphocytes in the local microenvironment as previously described^43^.

Interestingly, while homeostatic IL-12 production significantly promoted T-bet^high^ MP differentiation, the effect was partial with ~50% of the process occurring independently of IL-12 (Fig. 2b). By the same token, we found that the maintenance of T-bet^high^ MP subpopulation appears to be IL-12-independent (Fig. 4b). Because T-bet is induced and maintained through both IL-12-dependent and -independent pathways in the presence of TCR and costimulatory signals in Th1-type immune responses^44–46^, it is possible that additional cytokines may play a role in promoting optimal T-bet expression in steady state. Since IFN-γ had no clear effects on T-bet^high^ MP differentiation, type I IFN and IL-27^46^ are possible alternatives.

How CD4^+^ T lymphocytes distinguish basal IL-12 signals from the induced IL-12 response that triggers Th1-type immunity is an interesting question. One of the factors thought to prevent development of overt Th1 immune responses under steady-state conditions is the absence of strong TCR and costimulatory signals because these are induced significantly only under infectious and inflammatory conditions. In addition, it is possible that the level of homeostatic IL-12 does not exceed the threshold for naïve CD4^+^T cells to mount an overt Th1 response but is sufficient to maintain basal MP differentiation.

Our findings reveal CD8α^+^ DC1 as a major source of tonic IL-12 production. The DC1 subset is specialized for cross presentation and CD8^+^ T-cell response generation which are important for infectious and tumor immunity^29,47^. In addition, we and other groups have shown that the BatF3-sensitive DC subset regulates CD4^+^ T-cell development via IL-12 signaling in *Toxoplasma gondii* and other infections^30,48,49^. Consistent with the latter concept, we observed that under noninfectious conditions the differentiation of T-bet^high^ MP CD4^+^ T cells, in contrast to the total MP population, is strikingly promoted by the DC1 subset. This indicates that generation of MP cells and their differentiation into different subsets are governed by distinct mechanisms and implies that MP generation, which requires Ag recognition by naïve precursors^2^, does not depend on recognition of Ags presented on DC1 despite their MHCII expression. Although the antigen-presenting cell subpopulation that generates MP cells remains to be identified, it is thought to function by providing signals 1 and 2 to naïve T cells while the role of DC1 appears to be mainly as a source of signal 3 for T-bet^high^ MP cell differentiation.

TLR-MyD88 signaling driven by microbial-derived products is known to play a major role in the induction of IL-12^38,50^. Similarly, in our experiments MyD88 signaling and several TLRs were found to regulate steady-state IL-12 production by CD8α^+^ DCs. However, sensing of foreign-derived molecular signals was surprisingly dispensable for this process. Thus, GF mice that lack commensal microbiota as well as AF animals which are generated by feeding GF mice a chemically defined diet and housing them in sterile cages free of microbial contamination^6^ displayed splenic p40 production and T-bet^high^ MP levels comparable to those seen in SPF mice. These observations suggest that TLR triggering by self-derived products may be sufficient to provide the stimulus required for tonic IL-12 production by CD8α^+^ DCs and could contribute to this response under SPF conditions. Several endogenous MyD88-dependent TLR activators have been reported, which could exhibit this agonist activity. For example, HMGB1, β-defensin, surfactant protein A and D, and serum amyloid A are known to stimulate TLR2^51^. Regarding activators of Unc93b1-dependent TLRs, endogenous RNA activates TLR7 whereas IgG-chromatin complexes and mitochondrial DNA stimulate TLR9^51^. The involvement of these nucleic acid-sensing TLRs in upregulation of IL-12 is of particular interest because TLR7 and TLR9 are reported to recognize components of endogenous retroviruses in steady state to control their replication in C57BL/6 mice^52^.

CD40-mediated signaling has been shown to play a major role in the amplification of IL-12 responses triggered by microbial stimulation^22^. In this process CD40L expressed by activated CD4^+^ T lymphocytes significantly enhanced IL-12 production by CD40^+^ DCs. Consistent with this mechanism, we observed that CD40L amplifies IL-12 production by activated DC1 that have been basally primed by MyD88-activating agonists in steady state. Since in the absence of foreign stimulation CD40L levels are maintained at a normal level, tonic expression of this molecule may represent an endogenous mechanism for sustaining T-bet^high^ MP differentiation.

Because the generation of MP cells appears to depend on self Ag recognition, it has been speculated that this lymphocyte subpopulation could play a role as effectors of autoimmune disease^10,53^. Indeed, CD4^+^ T lymphocytes with an activated phenotype have been detected in the presumably foreign Ag-free environment of human cord blood and fetal tissues^54,55^, suggesting the existence of MP-like cells in humans. Whether this population is expanded in postnatal autoimmune states remains to be determined. Regardless, our murine studies suggest that while T-bet^high^ MP cell differentiation is promoted by homeostatic IL-12, maintenance of the same MP subpopulation is tightly regulated with IL-12 failing to impact its persistence in steady state (Fig. 4a, b). Moreover, activation of T-bet^high^ MP cells is induced only when they are exposed to high levels of IL-12 p70 (Fig. 1f and ref. ^2^). This refractory state may reflect the need to inhibit the expansion of self-reactive T-bet^high^ MP cells which putatively could have autoreactive effects.

Is MP differentiation entirely self-driven? Our findings indicate that MP cells can develop without the need for recognition of foreign Ags (Fig. 7c and ref. ^2^) and differentiate into the T-bet^high^ subset via IL-12 in the absence of microbial-derived products. Nevertheless, the strength of TCR affinity to self Ags may not be the only determinant regulating their differentiation. This possibility is supported by several findings. Thus, CD5 levels on CD4^+^ T lymphocytes only minimally correlate with the number of MP cells generated and fail to associate with their CD40L expression on a per-cell basis (Fig. 7c, d and ref. ^2^). In addition, we observed that naïve but not T-bet^−^ or T-bet^low^ MP cells can differentiate into T-bet^high^ cells (Figs. 3d and 4e), suggesting that the window period for T cells to receive IL-12 and convert to the T-bet^high^ subset is limited and that the exposure of precursors to the cytokine in this short period of time may be the critical determinant of whether or not they acquire the T-bet^high^ phenotype. Finally, regulatory T cells may influence MP generation through the effects on DC activation^10,53^. These additional mechanisms may limit the dependence of T-bet^high^ MP generation on self TCR affinity and may have evolved as further checkpoints preventing MP cells from triggering autoimmunity.

## Methods

### Mice

C57BL/6 CD45.2^+^ WT mice were purchased from Taconic Biosciences (Rensselaer, NY). C57BL/6 CD45.1^+^ WT, *Rag1* KO, CD45.2^+^ T-bet-ZsGreen reporter, *Rag2* / *Il2rg* DKO, *Il12b* KO, *Batf3* KO, *Il1r1* KO, OT-II *Rag1* KO, *Ifng* KO, *Tlr3* KO, C57BL/10 *Rag2* KO, and Marilyn *Rag2* KO mice were obtained from the National Institute of Allergy and Infectious Diseases (NIAID) contract facility at Taconic Biosciences. *Il12a* KO (002692), *Cd40l* KO (002770), and IFN-γ-YFP reporter (017581) mice were purchased from the Jackson Laboratory (Bar Harbor, ME). *Il23a* KO mice were obtained from Mutant Mouse Regional Resource Center. T-bet-AmCyan RORγt-E2Crimson double reporter and *Tlr11*/*12* KO mice are previously described^38,56–58^. For generation of CD4CreERT2 TCRα^flox^ IFN-γ-YFP T-bet-AmCyan mice, TCRα^flox^ mice^25^ provided by K. Rajewsky (Max Delbruck Center, Berlin, Germany) via A. Y. Rudensky (Memorial Sloan Kettering Cancer Center, New York, NY) to W. E. Paul were bred sequentially onto CD4CreERT2 (022356, Jackson Laboratory) and IFN-γ-YFP T-bet-AmCyan double reporter strains^2^. For generation of IL-12B p40-YFP reporter *Rag1* KO mice, IL-12B p40-YFP reporter mice^42^ provided by R. M. Locksley (University of California, San Francisco, CA) were crossed onto *Rag1* KO strain. *Myd88*, *Tlr2*, and *Tlr4* KO mice^59–61^ were provided by S. Akira (Osaka University, Osaka, Japan) via D. T. Golenbock (University of Massachusetts Medical School, Worcester, MA). *Tlr2*, *Tlr4*, and *Il18r1* KO mice were obtained from breeding stock maintained at the National Cancer Institute (NCI). *Tlr7* KO and *Tlr9* KO mice^62,63^ were provided by R. A. Flavell (Yale University School of Medicine, New Haven, CT) and S. Akira, respectively, via S. Bolland (NIAID, NIH). *Unc93b1*-mutant 3D mice^34^ were provided by B. Beutler (University of Texas Southwestern Medical Center, Dallas, TX) via D. T. Golenbock. P25 *Rag1* KO mice were obtained by crossing P25 TCR-Tg mice recognizing Ag85b of *Mycobacterium tuberculosis*^64^ provided by K. Takatsu (University of Tokyo, Tokyo, Japan) via J. D. Ernst (New York University, New York, NY) with *Rag1* KO mice. All mice were maintained in SPF animal facilities (ambient temperature 22 ± 3 °C, humidity 50 ± 20%, a light/dark cycle 14/10 h) in the NIAID, NCI, NIH, or Tohoku University Graduate School of Medicine except for GF and AF mice, which were bred and maintained in the NIAID Microbiome Program Gnotobiotic Animal Facility or the animal facility of Pohang University of Science and Technology as previously described^6^. All mice were used at the age of 8–16 weeks. In experiments analyzing KO animals, WT mice of the same gender and genetic background as KO mice were used as controls and maintained in adjacent caging in a room with standardized commensal flora. The care and handling of the animals used in our studies, including euthanasia, were in accordance with the animal study protocols approved by the NIAID or NCI Animal Care and Use Committee, by the Institutional Animal Care and Use Committees of the Pohang University of Science and Technology, or by the Institutional Committee for the Use and Care of Laboratory Animals of Tohoku University.

### In vivo chemical and mAb treatment

To activate CreERT2 recombinases, mice received two intraperitoneal injections at a 48 h interval of TMX (20 mg/mouse) dissolved in corn oil (both Sigma-Aldrich (St. Louis, MO)). To block IL-12 signaling in pre-existing MP cells, anti-IL-12B p40 mAb (C17.8, BE0051, 300 μg/20 g body weight) or control IgG (both from BioXCell (West Lebanon, NH)) were administered every other day for 1 week.

### Murine infection with *Toxoplasma gondii*

Tissue cysts of the Type II avirulent strain ME49 of *T. gondii* were obtained from the brains of chronically infected C57BL/6 WT mice as described previously^2^. For experimental infection, mice were inoculated by the intraperitoneal route with ~15 cysts per animal with one group of mice injected intraperitoneally with anti-MHCII mAb (Y3P, BE0178, 300 μg/20 g body weight, BioXCell) every three days starting on the day of infection.

### Adoptive transfer

Total CD4^+^ T lymphocytes were obtained from pooled splenocytes and lymph node cells of donor mice using a CD4^+^ T Cell Isolation Kit or CD4 Microbeads (Miltenyi Biotec (Bergisch Gladbach, Germany)). Purity was >90%. In some experiments, naïve CD4^+^ T cells were further purified by sorting for CD4^+^CD25^−^CD44^low^CD62L^high^ cells using a FACSAria II (BD Biosciences (San Jose, CA)). To obtain T-bet^high^, T-bet^low^, and T-bet^−^ MP cells, ZsGreen^high^, ZsGreen^low^, and ZsGreen^−^ subpopulations among CD4^+^CD25^−^CD44^high^CD62L^low^CD1d-tetramer^−^ cells were sorted from T-bet-ZsGreen reporter donor mice. Purity was >96%. Depending on the experiment, 5 × 10^5^–3 × 10^6^ donor cells were intravenously injected into recipient animals.

### Intracellular cytokine detection

For detection of IL-12 p40 and p35, cell suspensions of splenocytes were incubated in RPMI complete media for 6 h at 37 °C in the presence of Brefeldin A (1 μg/mL; BioLegend (San Diego, CA)). Cells were then harvested and subjected to intracellular staining as described below.

### Flow cytometric analysis

Single-cell suspensions were prepared from spleens and red blood cells lysed in ACK buffer. In some experiments, splenic cells were further enriched for CD4^+^ T lymphocytes using a CD4^+^ T Cell Isolation Kit or CD4 Microbeads (Miltenyi Biotec). Cells were suspended in staining buffer (PBS supplemented with 2% FBS) and incubated with CD16/32 mAb (2.4G2; Harlan Bioproducts (Indianapolis, IN)) for 10 min on ice. For negative control staining, the staining buffer was added with fluorochrome-unconjugated mAb and incubated for 1 h on ice. Cells were then incubated with either or a combination of the following mAbs for 20 min on ice: CD4 (RM4-5, 47-0042-82), CD8 (53-6.7, 56-0081-82), CD11b (M1/70, 25-0112-82), CD19 (1D3, 12-0193-82), CD40L (MR1, 12-1541-82), CD44 (IM7, 48-0441-82), CD45R/B220 (RA3-6B2, 64-0452-82), CD62L (MEL-14, 83-0621-42), anti-I-A/I-E (M5/114.15.2, 48-5321-82), anti-NK1.1 (PK136, 12-5941-82), anti-TCRβ (H57-597, 45-5961-82) (ThermoFisher Scientific (Waltham, MA)), CD3 (17A2, 100218), CD5 (53-7.3, 100626), CD11c (N418, 117310), CD25 (PC61, 102016), CD40 (3/23, 124630), CD45.1 (A20, 110743), CD45.2 (104, 109822), CD86 (GL-1, 105028), and anti-CXCR3 (CXCR3-173, 126512) (BioLegend). In some experiments, cells were stained with CD1d-tetramer (PBS-57) obtained from the NIH Tetramer Core Facility (Emory University, Atlanta, GA) to exclude NKT cells. To detect intracellular products, cells were fixed and permeabilized using Foxp3/Transcription Factor Staining Buffer Set for 30 min on ice after surface staining and then stained with mAbs against Foxp3 (FJK-16s, 25-5773-82), IL-12A p35 (27537, MA5-23559), and/or IL-12B p40 (C17.8, 53-7123-82) (ThermoFisher Scientific) for 20 min on ice. For T-bet detection, fixed cells were stained with anti-T-bet (O4-46, 561268, BD Biosciences) mAb for 2 h at room temperature. All mAbs were used at a 1:100 dilution except in the case of anti-T-bet where the mAb was used at a 1:17 dilution. Flow cytometry was performed using either LSR II, Fortessa, or Symphony cytometers (BD Biosciences) and the data analyzed with FlowJo software (TreeStar (Ashland, OR)). The gating strategy for detection of MP CD4^+^ T lymphocytes and DC1 is summarized in Supplementary Fig. 9.

### Cytokine measurement by ELISA

Sorted DC1, DC2, and DN DC were cultured in RPMI complete medium in 96-well plates for 24 h at 37 °C (1 × 10^6^ cells/mL). Cells were then centrifuged, and IL-12 p40 and p70 levels in the culture supernatants measured using Mouse Quantikine ELISA Kits (R&D Systems (Minneapolis, MN)).

### Immunofluorescent staining and confocal imaging

Excised spleens were incubated in a Fixation and Permeabilization Solution (554722, BD Biosciences) diluted in PBS overnight followed by dehydration in 30% sucrose prior to embedding in OCT Compound (Sakura Finetek (Torrance, CA)). Eighteen-micron sections were cut on a CM3050S cryostat (Leica (Wetzlar, Germany)) and adhered to Superfrost Plus slides (VWR (Radnor, PA)). Frozen sections were further permeabilized and blocked in PBS containing 0.3% Triton X-100 (Sigma-Aldrich) and 1% normal sera from mouse, rat, and rabbit (Jackson Immunoresearch (West Grove, PA)) followed by staining with mAbs diluted in blocking buffer. The mAbs used were directed against: B220 (RA3-6B2, 56-0452-82), CD4 (RM4-5, 48-0042-82), GFP/YFP (A31851) (ThermoFisher Scientific), CD8 (53-6.7, 100758), and CD11c (N418, 117311) (Biolegend). After staining, slides were mounted with Fluormount G (Southern Biotech (Birmingham, AL)) and examined on a Leica TCS SP8 confocal microscope. Images were analyzed by Imaris software (Bitplane (Belfast, United Kingdom)).

### Statistical analysis

In survival experiments, a log-rank test was used to establish statistical significance. In all other instances, a Student’s *t* test was employed. *p* values <0.05 were considered significant.

### Reporting summary

Further information on research design is available in the Nature Research Reporting Summary linked to this article.

## Supplementary information

Supplementary Information

Reporting Summary

## Data Availability

All data are included in the article and supplementary information files or available from the authors upon reasonable requests. The source data underlying Figs. 1f, h, 2a–g, 3, 4a, b, d, 5, 6, 7c–i, 8, Supplementary Figs. 1c, d, 2b, 3, 4a, c, 5–7, 8a, d are provided as a Source Data file.

## Source data

Source Data
