# Author Correction: Requirements for the differentiation of innate T-bet^high^ memory-phenotype CD4^+^ T lymphocytes under steady state

**DOI:** 10.1038/s41467-020-18568-5

**Published:** 2020-09-10

**Authors:** Takeshi Kawabe, Jaeu Yi, Akihisa Kawajiri, Kerry Hilligan, Difeng Fang, Naoto Ishii, Hidehiro Yamane, Jinfang Zhu, Dragana Jankovic, Kwang Soon Kim, Giorgio Trinchieri, Alan Sher

**Affiliations:** 1 Immunobiology Section, Laboratory of Parasitic Diseases, National Institute of Allergy and Infectious Diseases (NIAID), National Institutes of Health (NIH), Bethesda, MD 20892 USA; 2 Department of Microbiology and Immunology, Tohoku University Graduate School of Medicine, Sendai, 980-8575 Japan; 3 Academy of Immunology and Microbiology, Institute for Basic Science, Pohang, 37666 Republic of Korea; 4 Department of Integrative Biosciences and Biotechnology, Pohang University of Science and Technology, Pohang, 37673 Republic of Korea; 5 Immune Cell Biology Programme, Malaghan Institute of Medical Research, Wellington, 6012 New Zealand; 6 Molecular and Cellular Immunoregulation Section, Laboratory of Immune System Biology, NIAID, NIH, Bethesda, MD 20892 USA; 7 Cellular Immunology Section, Vaccine Research Center, NIAID, NIH, Bethesda, MD 20892 USA; 8 Cancer and Inflammation Program, Center for Cancer Research, National Cancer Institute, NIH, Bethesda, MD 20892 USA

**Keywords:** Immunological memory, Infection, Innate immune cells, CD4-positive T cells

Correction to: *Nature Communications* 10.1038/s41467-020-17136-1, published online 06 July 2020.

The original version of this Article contained an error in the author affiliations.

The affiliation of Kerry Hilligan with ‘Immune Cell Biology Programme, Malaghan Institute of Medical Research, Wellington 6012, New Zealand’ was inadvertently omitted.

This has now been corrected in both the PDF and HTML versions of the Article.

