# Supplementary material for: Requirements for the differentiation of innate T-bethigh memory-phenotype CD4+ T lymphocytes under steady state
Source: Nat Commun. 2020 Jul 6;11:3366. doi: 10.1038/s41467-020-17136-1 (PMC7338451; doi:10.1038/s41467-020-17136-1)
Supplement: Supplementary file 1 — Supplementary Information [file 41467_2020_17136_MOESM1_ESM.pdf]

Kawabe, et al.

Requirements for the differentiation of innate T-bet^{high} memory-phenotype CD4^{+} T lymphocytes under steady state

Supplementary information

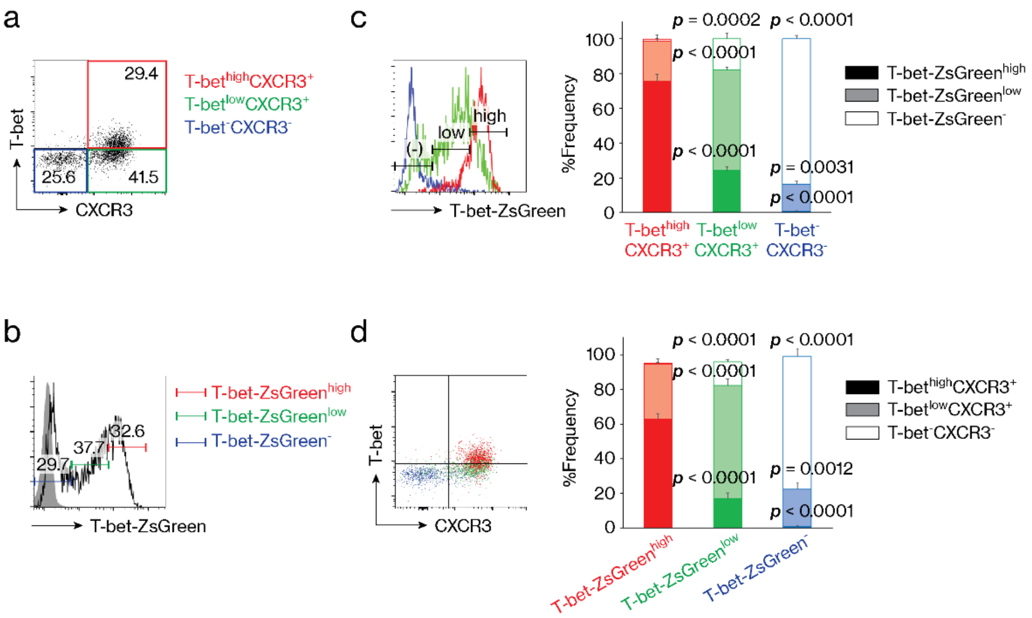

Supplementary Fig. 1. ZsGreen expression levels on T-bet^{high}CXCR3^{+}, T-bet^{low}CXCR3^{+}, and T-bet^{-}CXCR3^{-} subsets in T-bet-ZsGreen reporter mice. (**a** and **b**) The representative dot plot and histogram show (a) T-bet and CXCR3 and (b) ZsGreen expression in MP CD4^{+} T lymphocytes from one of five T-bet-ZsGreen reporter mice analyzed. (**c**) The histogram depicts ZsGreen expression in the indicated subsets of MP cells from a representative T-bet-ZsGreen reporter mouse while the bar graph shows the frequency (mean ± SD) of ZsGreen^{high}, ZsGreen^{low}, and ZsGreen^{-} fraction among each MP subset (n=5 mice). (**d**) The dot plot displays T-bet and CXCR3 expression in the indicated subset of MP cells from the same representative reporter mouse while the bar graph depicts the fraction (mean ± SD) of T-bet^{high}CXCR3^{+}, T-bet^{low}CXCR3^{+}, and T-bet^{-}CXCR3^{-} among each MP subset (n=5 mice). Data shown are pooled from 2 independent experiments performed. Red T-bet^{high}CXCR3^{+} / ZsGreen^{high}, Green T-bet^{low}CXCR3^{+} / ZsGreen^{low}, Blue T-bet^{-}CXCR3^{-} / ZsGreen^{-}. A two-sided *t* test was performed to assess statistical significance. Source data are provided as a Source Data file.

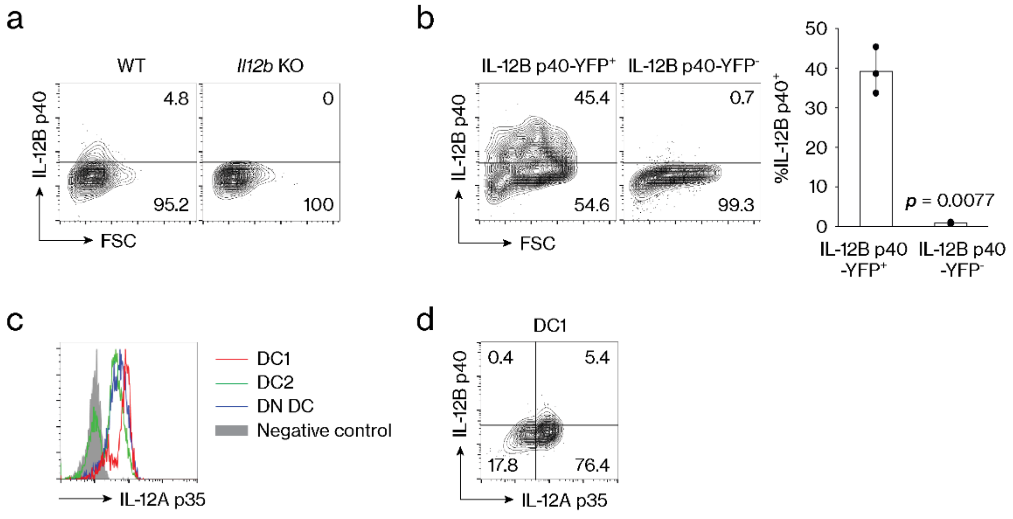

Supplementary Fig. 2. DC1 express both p40 and p35 in steady state. (**a**) The representative contour plots show p40 expression in CD8α+ DCs from WT and *Il12b* KO animals (n=5 mice). (**b**) The contour plots display p40 protein expression in YFP+ and YFP-CD8α+ DCs sorted from p40-YFP reporter mice while the bar graph shows the frequency (mean ± SD) of p40+ cells among each sorted cell population (n=3 mice). (**c**) The representative histogram depicts p35 expression in the indicated DC subsets (n=4 mice). Red DC1, green DC2, blue DN DC. (**d**) The representative plot shows expression of p40 and p35 in CD8α+ DCs (n=4 mice). Data shown are representative of 2 independent experiments. A two-sided *t* test was performed to assess statistical significance. Source data are provided as a Source Data file.

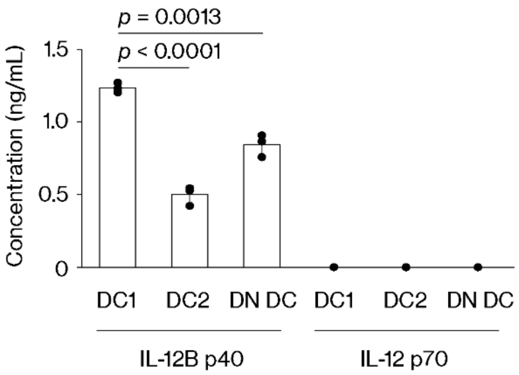

Supplementary Fig. 3. Production of IL-12 p40 versus p70 by DC subsets. The bar graph indicates the concentration (mean ± SD) of p40 and p70 produced by the indicated DC subsets (n=3 mice). Data shown are representative of 2 independent experiments. A two-sided *t* test was performed to assess statistical significance. Source data are provided as a Source Data file.

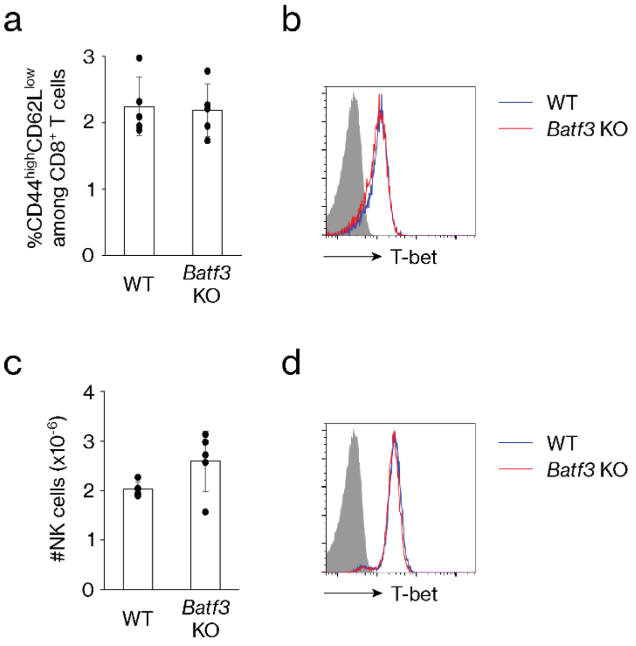

Supplementary Fig. 4. Deficiency in BatF3 does not affect T-bet expression in CD8^{+} T or NK cells. (**a** and **b**) The bar graph shows the frequency (mean ± SD) of CD44^{high}CD62L^{low} cells among CD8^{+} T lymphocytes from WT and *Batf3* KO animals while the representative histogram indicates T-bet expression in the CD44^{high}CD62L^{low}CD8^{+} T cell subpopulation from each group (n=5 mice). (**c** and **d**) The graph displays the number (mean ± SD) of NK cells from each group and the histogram depicts T-bet expression in the same cell population (n=5 mice). Blue and red lines indicate WT and *Batf3* KO mice, respectively. Filled histograms show negative control staining. Data are representative of 2 independent experiments. Source data are provided as a Source Data file.

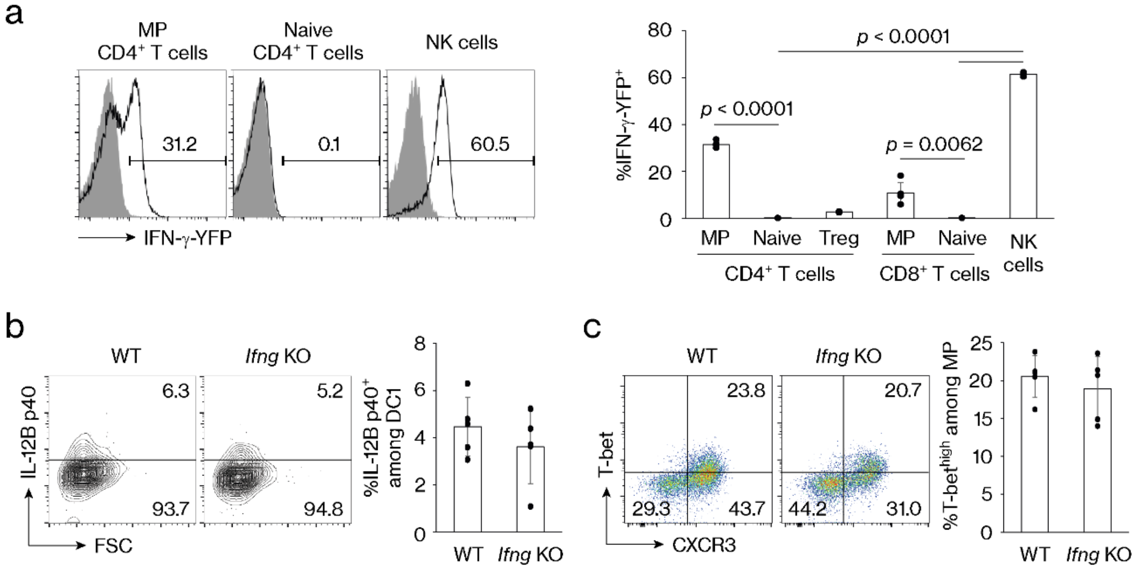

Supplementary Fig. 5. Tonic IFN-γ is dispensable for enhancement of both DC1 p40 expression and MP differentiation. (**a**) The representative histograms display YFP expression in the indicated cell populations from IFN-γ-YFP reporter animals while the bar graph indicates the YFP^+ fraction (mean ± SD) among each cell population (n=5 mice). Filled histograms show negative controls from non-reporter mice. (**b**) The contour plots depict p40 expression in $CD8\alpha^+$ DCs from WT and *Ifng* KO mice and the graph shows the frequency (mean ± SD) of $p40^+$ cells among the same DC subset from each group (n=5 mice). (**c**) The representative dot plots display expression of T-bet and CXCR3 in MP cells from the indicated animals while the bar graph shows the frequency (mean ± SD) of $T\text{-}bet^{high}CXCR3^+$ cells among the same MP populations from each group (n=5 mice). Data shown are representative of 2 independent experiments performed. A two-sided *t* test was performed to assess statistical significance. Source data are provided as a Source Data file.

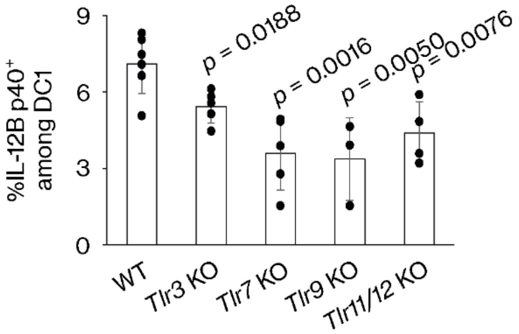

Supplementary Fig. 6. Effects of different TLR deficiencies on tonic p40 production by DC1. The bar graph shows the frequency (mean ± SD) of p40^{+} cells among DC1 from WT as well as indicated KO mice (WT n=6 mice; *Tlr3* KO n=5 mice; *Tlr7* KO n=5 mice; *Tlr9* KO n=3 mice; *Tlr11/12* KO n=4 mice). Data are pooled from 2 independent experiments performed. A two-sided *t* test was performed to assess statistical significance. Source data are provided as a Source Data file.

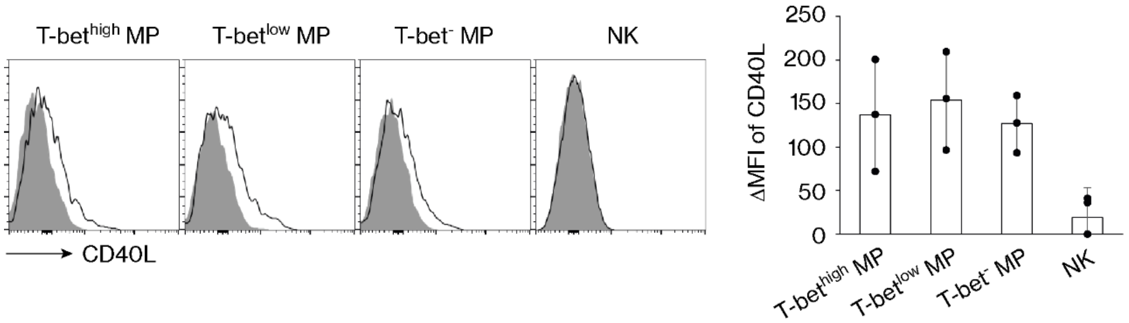

Supplementary Fig. 7. MP cells homeostatically express CD40L regardless of their T-bet levels. The representative histograms display CD40L expression in the indicated cell populations obtained from T-bet-ZsGreen reporter mice while the bar graph shows ΔMFI (mean ± SD) of CD40L expression in each cell population (n=3 mice). Source data are provided as a Source Data file.

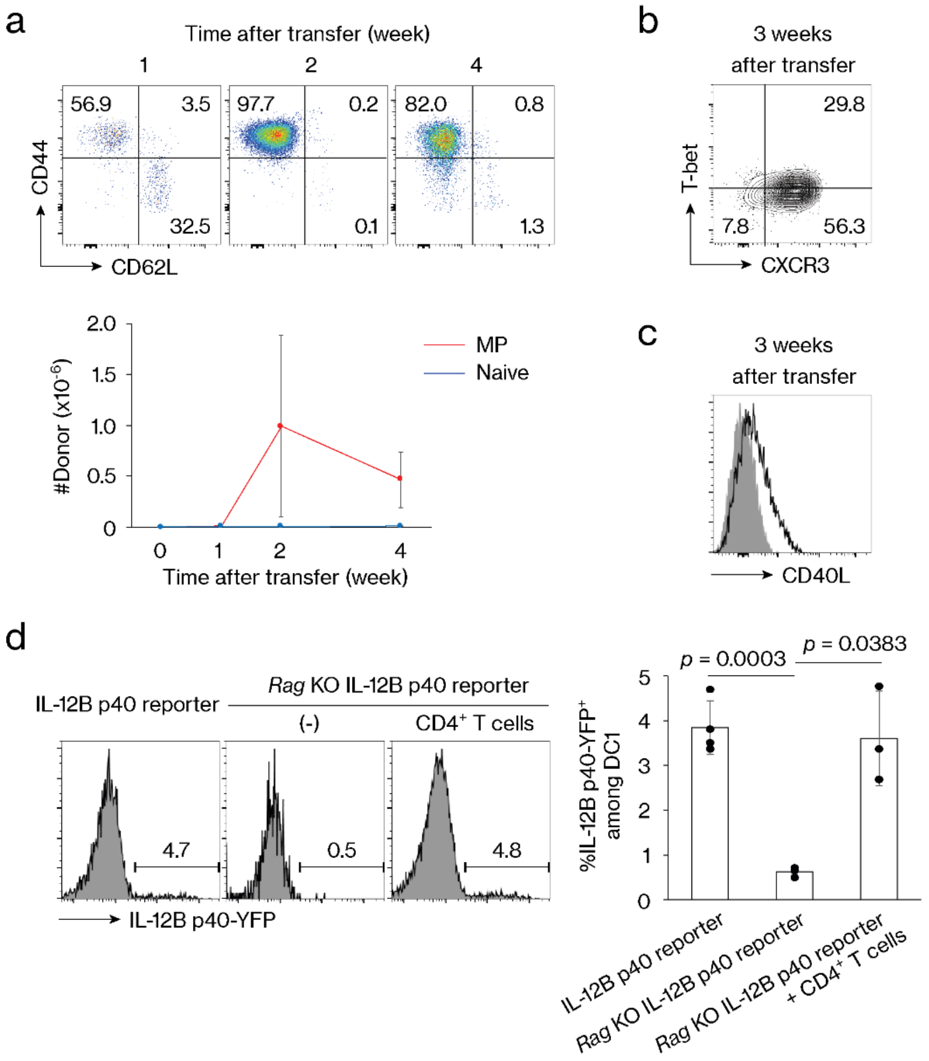

Supplementary Fig. 8. CD4$^+$ T cells acquire an MP-like phenotype and upregulate DC1 p40 levels in *Rag* KO recipients. (**a – c**) CD4$^+$ T lymphocytes were transferred into *Rag* KO animals which were then recovered and analyzed 1, 2, and 4 weeks later. (a) The representative dot plots show CD44 and CD62L expression in the recovered donor cell populations while the graph displays the number (mean ± SD) of recovered donor cells at the indicated week(s) after transfer (Week 1 n=4 mice; Week 2 n=5 mice; Week 4 n=4 mice). Red and blue lines show CD44^{high}CD62L^{low} and CD44^{low}CD62L^{high} donor cells, respectively. Data are pooled from 2 independent experiments performed.

(b) T-bet and CXCR3 expression in CD44^{high}CD62L^{low} donor cells 3 weeks after transfer is depicted (representative data from 4 animals). (c) CD40L levels on CD44^{high}CD62L^{low} donor cells 3 weeks after transfer are displayed (representative of 3 mice). The filled histogram shows negative control staining. (**d**) The same transfer described above was performed using *Rag* KO p40-YFP reporter animals as recipients. Untransferred WT and *Rag* KO p40-YFP reporter mice were also analyzed. The representative histograms show YFP expression in the recipient CD8α^+ DCs 3 weeks after transfer while the bar graph indicates the frequency (mean ± SD) of YFP$^+$ cells among CD8α^+ DCs from each group (IL-12B p40 reporter n=4 mice; *Rag* KO IL-12B p40 reporter n=3 mice; *Rag* KO IL-12B p40 reporter + CD4$^+$ T cells n=3 mice). Data are representative of 2 independent experiments. A two-sided *t* test was performed to assess statistical significance. Source data are provided as a Source Data file.

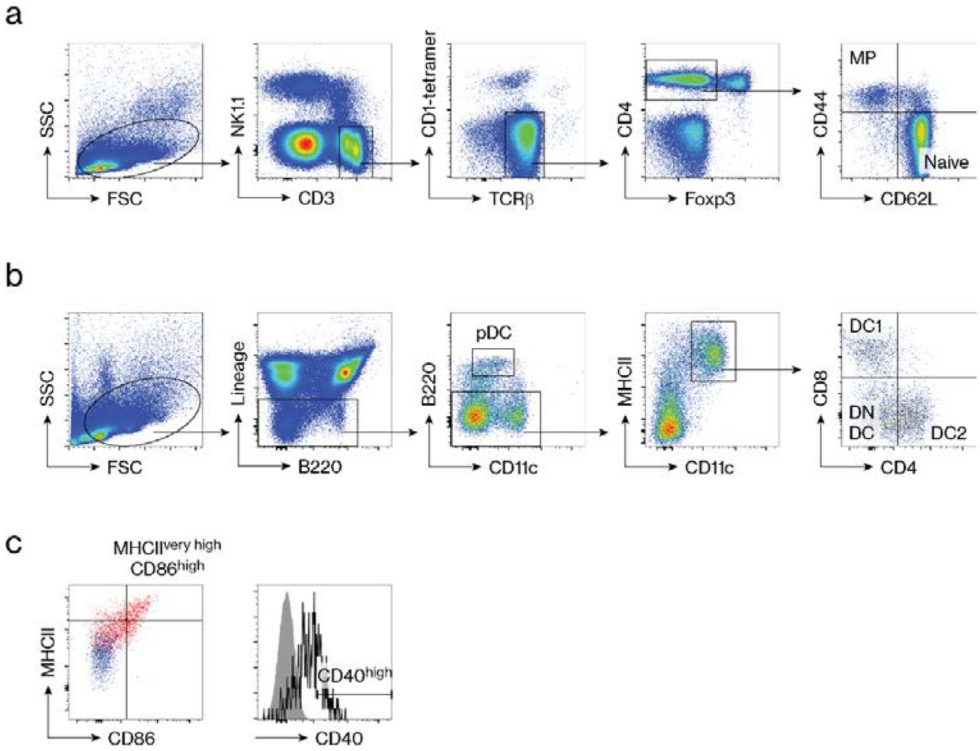

Supplementary Fig. 9. Gating strategy for flow cytometric analyses. (**a**) To detect MP and naïve $CD4^+$ T cells (Fig. 1, 2a-c, 3, 4, 5b-d, g, j, 6a, c, f, 7a-e, g, h, 8b, d, e, Supplementary Fig. 1, 4, 5c, 7, 8a-c), total singlet cells were gated for $CD3^+NK1.1^-$ $TCR\beta^+$CD1-$tetramer^-Foxp3^-CD4^+$ population. MP and naïve cells were then defined as $CD44^{high}CD62L^{low}$ and $CD44^{low}CD62L^{high}$ subpopulations, respectively. (**b**) For conventional and plasmacytoid DC detection (Fig. 2d, e, 5a, f, h, 6b, e, 7f, i, 8a, c, Supplementary Fig. 2, 3, 5b, 6, 8d), total singlet cells were gated for Lineage $(CD3/CD19/NK1.1)^-B220^-CD11c^+MHCII^+$ and $Lineage^-B220^+CD11c^{int}CD11b^-$ populations, respectively. Conventional DCs were further classified into $CD8\alpha^+$ DC1, $CD4^+$ DC2, and $CD8\alpha^-CD4^-$ DN DC subsets. (**c**) For examination of activation status of DCs (Fig. 2f, g, 5e, i, 6d), expression levels of MHCII, CD86, and CD40 were measured. $MHCII^{very\ high}CD86^{high}CD40^{high}$ cells in $CD8\alpha^+$ DCs (red dots and open histogram) were analyzed using plasmacytoid DC (blue dots) and T lymphocyte (filled histogram) populations as the reference.
