# Supplementary material for: Requirements for the differentiation of innate T-bethigh memory-phenotype CD4+ T lymphocytes under steady state
Source: Nat Commun. 2020 Jul 6;11:3366. doi: 10.1038/s41467-020-17136-1 (PMC7338451; doi:10.1038/s41467-020-17136-1)
Supplement: Supplementary file 2 — Reporting Summary [file 41467_2020_17136_MOESM2_ESM.pdf]

nature research

Corresponding author(s): Takeshi Kawabe
Alan Sher

Last updated by author(s): Jun 7, 2020

# Reporting Summary

Nature Research wishes to improve the reproducibility of the work that we publish. This form provides structure for consistency and transparency in reporting. For further information on Nature Research policies, see Authors & Referees and the Editorial Policy Checklist.

## Statistics

For all statistical analyses, confirm that the following items are present in the figure legend, table legend, main text, or Methods section.

| n/a | Confirmed | |
|---|---|---|
| ☐ | ☒ | The exact sample size (*n*) for each experimental group/condition, given as a discrete number and unit of measurement |
| ☐ | ☒ | A statement on whether measurements were taken from distinct samples or whether the same sample was measured repeatedly |
| ☐ | ☒ | The statistical test(s) used AND whether they are one- or two-sided<br>*Only common tests should be described solely by name; describe more complex techniques in the Methods section.* |
| ☒ | ☐ | A description of all covariates tested |
| ☒ | ☐ | A description of any assumptions or corrections, such as tests of normality and adjustment for multiple comparisons |
| ☐ | ☒ | A full description of the statistical parameters including central tendency (e.g. means) or other basic estimates (e.g. regression coefficient) AND variation (e.g. standard deviation) or associated estimates of uncertainty (e.g. confidence intervals) |
| ☐ | ☒ | For null hypothesis testing, the test statistic (e.g. *F*, *t*, *r*) with confidence intervals, effect sizes, degrees of freedom and *P* value noted<br>*Give P values as exact values whenever suitable.* |
| ☒ | ☐ | For Bayesian analysis, information on the choice of priors and Markov chain Monte Carlo settings |
| ☒ | ☐ | For hierarchical and complex designs, identification of the appropriate level for tests and full reporting of outcomes |
| ☐ | ☒ | Estimates of effect sizes (e.g. Cohen's *d*, Pearson's *r*), indicating how they were calculated |

*Our web collection on statistics for biologists contains articles on many of the points above.*

## Software and code

Policy information about availability of computer code

| | |
|---|---|
| Data collection | FACSDiva 8 (BD Biosciences) |
| Data analysis | FlowJo 10 (BD Biosciences), Imaris 9 (Bitplane) |

For manuscripts utilizing custom algorithms or software that are central to the research but not yet described in published literature, software must be made available to editors/reviewers. We strongly encourage code deposition in a community repository (e.g. GitHub). See the Nature Research guidelines for submitting code & software for further information.

## Data

Policy information about availability of data

All manuscripts must include a data availability statement. This statement should provide the following information, where applicable:
- Accession codes, unique identifiers, or web links for publicly available datasets
- A list of figures that have associated raw data
- A description of any restrictions on data availability

All data are included in the article and supplementary information files or available from the authors upon reasonable requests. The source data underlying Figs. 1f, h, 2a-g, 3, 4a, b, d, 5, 6, 7c-i, 8, Supplementary Figs. 1c, d, 2b, 3, 4a, c, 5-7, 8a, d are provided as a Source Data file.

# Field-specific reporting

Please select the one below that is the best fit for your research. If you are not sure, read the appropriate sections before making your selection.

☒ Life sciences ☐ Behavioural & social sciences ☐ Ecological, evolutionary & environmental sciences

# Life sciences study design

All studies must disclose on these points even when the disclosure is negative.

| | |
|---|---|
| Sample size | Animal sample size was determined based on both our previous work using similar models (Kawabe T, et al. Sci Immunol 2(12): eaam9304, 2017) and experimental feasibility. |
| Data exclusions | No data were excluded. |
| Replication | All experiments were performed at least twice and similar results obtained. Details are shown in the figure legends. |
| Randomization | Animal experiments were not randomized. Animals of similar age and sex were used to control for covariates. For comparison between KO and WT animals, WT mice with the same genetic background as KO mice were used as controls and maintained in adjacent caging in a room with standardized commensal flora. |
| Blinding | Investigators were not blinded because all samples were analyzed in the same way. |

# Reporting for specific materials, systems and methods

We require information from authors about some types of materials, experimental systems and methods used in many studies. Here, indicate whether each material, system or method listed is relevant to your study. If you are not sure if a list item applies to your research, read the appropriate section before selecting a response.

## Materials & experimental systems

| n/a | Involved in the study | |
|---|---|---|
| ☐ | ☒ | Antibodies |
| ☒ | ☐ | Eukaryotic cell lines |
| ☒ | ☐ | Palaeontology |
| ☐ | ☒ | Animals and other organisms |
| ☒ | ☐ | Human research participants |
| ☒ | ☐ | Clinical data |

## Methods

| n/a | Involved in the study | |
|---|---|---|
| ☒ | ☐ | ChIP-seq |
| ☐ | ☒ | Flow cytometry |
| ☒ | ☐ | MRI-based neuroimaging |

## Antibodies

Antibodies used

All mAbs used in this study, their clone numbers, catalog numbers, and validation are shown below. All mAbs were used at a 1:100 dilution for flow cytometric analysis except in the case of anti-T-bet where the mAb was used at a 1:17 dilution.

[ThermoFisher Scientific]
CD4: RM4-5, 47-0042-82, 48-0042-82, "tested by flow cytometric analysis of mouse splenocytes"
CD8: 53-6.7, 56-0081-82, "tested by flow cytometric analysis of mouse thymocyte and splenocyte suspensions"
CD11b: M1/70, 25-0112-82, "tested by flow cytometric analysis of mouse splenocytes and bone marrow cells"
CD19: 1D3, 12-0193-82, "tested by flow cytometric analysis of mouse splenocytes"
CD40L: MR1, 12-1541-82, "tested by flow cytometric analysis of stimulated splenocytes"
CD44: IM7, 48-0441-82, "tested by flow cytometric analysis of mouse splenocytes"
CD45R/B220: RA3-6B2, 64-0452-82, 56-0452-82, "tested by flow cytometric analysis of mouse splenocytes"
CD62L: MEL-14, 83-0621-42, "tested by flow cytometric analysis of mouse splenocytes"
Foxp3: FJK-16s, 25-5773-82, "tested by intranuclear staining and flow cytometric analysis using the Foxp3/Transcription Factor Buffer Set (00-5523) and protocol"
GFP/YFP: A31851, used at least in 8 studies as shown in "https://www.thermofisher.com/antibody/product/GFP-Antibody-Polyclonal/A-31851"
I-A/I-E: M5/114.15.2, 48-5321-82, "tested by flow cytometric analysis of mouse splenocytes"
IL-12A p35: 27537, MA5-23559, tested by intracellular staining and "flow cytometric analysis of mouse splenocytes treated with LPS" as shown in "https://www.thermofisher.com/antibody/product/IL-12-p35-Antibody-clone-27537-Monoclonal/MA5-23559"
IL-12B p40: C17.8, 53-7123-82, "tested by intracellular staining and flow cytometric analysis of stimulated mouse cells"
NK1.1: PK136, 12-5941-82, "tested by flow cytometric analysis of C57Bl/6 mouse splenocytes"
TCRb: H57-597, 45-5961-82, "tested by flow cytometric analysis of mouse splenocytes"

[Biolegend]
CD3: 17A2, 100218, tested by flow cytometric analysis of "C57BL/6 splenocytes"
CD5: 53-7.3, 100626, tested by flow cytometric analysis of "C57BL/6 mouse splenocytes"
CD8: 53-6.7, 100758, tested by immunohistochemical analysis of "C57BL/6 mouse frozen lymph node section fixed with 4% paraformaldehyde" as shown in "https://www.biolegend.com/it-it/products/alexa-fluor-594-anti-mouse-cd8a-antibody-9608"
CD11c: N418, 117310, 117311, tested by flow cytometric analysis of "C57BL/6 mouse splenocytes"
CD25: PC61, 102016, tested by flow cytometric analysis of "Con A-stimulated (2 days) BALB/c mouse splenocytes"
CD40: 3/23, 124630, tested by flow cytometric analysis of "C57BL/6 mouse splenocytes"

CD45.1: A20, 110743, tested by flow cytometric analysis of "SJL mouse splenocytes"
CD45.2: 104, 109822, tested by flow cytometric analysis of "C57BL/6 mouse splenocytes"
CD86: GL-1, 105028, tested by flow cytometric analysis of "LPS-stimulated (3 days) C57BL/6 mouse splenocytes"
CXCR3: CXCR3-173, 126512, tested by flow cytometric analysis of "C57BL/6 splenocytes"

[BD Biosciences]
T-bet: O4-46, 561268, tested by flow cytometric analysis of human "whole blood" as shown in "https://www.bdbiosciences.com/us/applications/research/t-cell-immunology/th-1-cells/intracellular-markers/cell-signalling-and-transcription-factors/human/pe-mouse-anti-t-bet-o4-46/p/561268"; We validated mouse reactivity by flow cytometric analysis of C57BL/6 splenocytes from T-bet-ZsGreen reporter mice as described in Fig. 1 and Supplementry Fig. 1.

[BioXCell]
IL-12B p40: C17.8, BE0051, used in more than 10 studies as shown in "https://bxcell.com/product/invivomab-anti-m-il-12-il-23/"; We also validated its effect in our previous study (Kawabe T, et al. Sci Immunol 2(12): eaam9304, 2017).
MHCII: Y3P, BE0178, used at least in 6 studies as shown in "https://bxcell.com/product/m-i-a/"; We also validated its effect in our previous study (Kawabe T, et al. Sci Immunol 2(12): eaam9304, 2017).

Validation

In the table above the validation method for each antibody is indicated in the last column. Validation was performed by the manufacturers and their statements are cited.

# Animals and other organisms

Policy information about studies involving animals; ARRIVE guidelines recommended for reporting animal research

Laboratory animals

C57BL/6 CD45.2+ WT mice were purchased from Taconic Biosciences (Rensselaer, NY). C57BL/6 CD45.1+ WT, Rag1 KO, CD45.2+ T-bet-ZsGreen reporter, Rag2 / Il2rg DKO, Il12b KO, Batf3 KO, Il1r1 KO, OT-II Rag1 KO, Ifng KO, Tlr3 KO, C57BL/10 Rag2 KO, and Marilyn Rag2 KO mice were obtained from the National Institute of Allergy and Infectious Diseases (NIAID) contract facility at Taconic Biosciences. Il12a KO (002692), Cd40l KO (002770), and IFN-g-YFP reporter (017581) mice were purchased from the Jackson Laboratory (Bar Harbor, ME). Il23a KO mice were obtained from Mutant Mouse Regional Resource Center. T-bet-AmCyan RORgt-E2Crimson double reporter and Tlr11 / Tlr12 DKO mice are previously described. For generation of CD4CreERT2 TCRa(flox) IFN-g-YFP T-bet-AmCyan mice, TCRa(flox) mice provided by K. Rajewsky (Max Delbruck Center, Berlin, Germany) via A. Y. Rudensky (Memorial Sloan Kettering Cancer Center, New York, NY) to W. E. Paul were bred sequentially onto CD4CreERT2 (022356, Jackson Laboratory) and IFN-g-YFP T-bet-AmCyan double reporter strains. For generation of IL-12B p40-YFP reporter Rag1 KO mice, IL-12B p40-YFP reporter mice provided by R. M. Locksley (University of California, San Francisco, CA) were crossed onto Rag1 KO strain. Myd88, Tlr2, and Tlr4 KO mice were provided by S. Akira (Osaka University, Osaka, Japan) via D. T. Golenbock (University of Massachusetts Medical School, Worcester, MA). Tlr2, Tlr4, and Il18r1 KO mice were obtained from breeding stock maintained at the National Cancer Institute (NCI). Tlr7 KO and Tlr9 KO mice were provided by R. A. Flavell (Yale University School of Medicine, New Haven, CT) and S. Akira, respectively, via S. Bolland (NIAID, NIH). Unc93b1-mutant 3D mice were provided by B. Beutler (University of Texas Southwestern Medical Center, Dallas, TX) via D. T. Golenbock. P25 Rag1 KO mice were obtained by crossing P25 TCR Tg mice recognizing Ag85b of Mycobacterium tuberculosis provided by K. Takatsu (University of Tokyo, Tokyo, Japan) via J. D. Ernst (New York University, New York, NY) with Rag1 KO mice. All mice were maintained in SPF animal facilities (ambient temperature 22 ± 3 C, humidity 50 ± 20 %, a light/dark cycle 14/10 hr) in the NIAID, NCI, NIH, or Tohoku University Graduate School of Medicine except for GF and AF mice, which were bred and maintained in the NIAID Microbiome Program Gnotobiotic Animal Facility or the animal facility of Pohang University of Science and Technology as previously described. All mice were used at the age of 8 – 16 wks. In experiments analyzing KO animals, wild-type mice with the same gender and genetic background as KO mice were used as controls and maintained in adjacent caging in a room with standardized commensal flora.

Wild animals

No wild animals were used.

Field-collected samples

No field-collected samples were used.

Ethics oversight

The care and handling of the animals used in our studies, including euthanasia, were in accordance with the animal study protocols approved by the NIAID or NCI Animal Care and Use Committee, by the Institutional Animal Care and Use Committees of the Pohang University of Science and Technology, or by the Institutional Committee for the Use and Care of Laboratory Animals of Tohoku University.

Note that full information on the approval of the study protocol must also be provided in the manuscript.

# Flow Cytometry

## Plots

Confirm that:

☒ The axis labels state the marker and fluorochrome used (e.g. CD4-FITC).

☒ The axis scales are clearly visible. Include numbers along axes only for bottom left plot of group (a 'group' is an analysis of identical markers).

☒ All plots are contour plots with outliers or pseudocolor plots.

☒ A numerical value for number of cells or percentage (with statistics) is provided.

## Methodology

| | |
|---|---|
| Sample preparation | Single cell suspensions were prepared from spleens and red blood cells lysed in ACK buffer. In some experiments, splenic cells were further enriched for CD4+ T lymphocytes using a CD4+ T Cell Isolation Kit or CD4 Microbeads (Miltenyi Biotec). |
| Instrument | BD Fortessa, Symphony, or LSR II. |
| Software | BD FACS Diva |
| Cell population abundance | For sorting experiments, purity of the samples was determined by post-sort analysis and was >96%. |
| Gating strategy | To detect MP and naïve CD4+ T cells (Fig. 1, 2a-c, 3, 4, 5b-d, g, j, 6a, c, f, 7a-e, g, h, 8b, d, e, Supplementary Fig. 1, 4, 5c, 7, 8a-c), total singlet cells were gated for CD3+ NK1.1- TCRb+ CD1-tetramer- Foxp3- CD4+ population. MP and naïve cells were then defined as CD44(high) CD62L(low) and CD44(low) CD62L(high) subpopulations, respectively. For conventional and plasmacytoid DC detection (Fig. 2d, e, 5a, f, h, 6b, e, 7f, i, 8a, c, Supplementary Fig. 2, 3, 5b, 6, 8d), total singlet cells were gated for Lineage (CD3/CD19/NK1.1)- B220- CD11c+ MHCII+ and Lineage- B220+ CD11c(int) CD11b- populations, respectively. Conventional DCs were further classified into CD8a+ DC1, CD4+ DC2, and CD8a- CD4- DN DC subsets. For examination of activation status of DCs (Fig. 2f, g, 5e, i, 6d), expression levels of MHCII, CD86, and CD40 were measured. MHCII(very high) CD86(high) CD40(high) cells in CD8a+ DCs were analyzed using plasmacytoid DC and T lymphocyte populations as the reference. Details are shown in Supplementary Fig. 9. |

☒ Tick this box to confirm that a figure exemplifying the gating strategy is provided in the Supplementary Information.
